# Neuropathy and neural plasticity in the subcutaneous white adipose depot

**DOI:** 10.1371/journal.pone.0221766

**Published:** 2019-09-11

**Authors:** Magdalena Blaszkiewicz, Jake W. Willows, Amanda L. Dubois, Stephen Waible, Kristen DiBello, Lila L. Lyons, Cory P. Johnson, Emma Paradie, Nicholas Banks, Katherine Motyl, Merilla Michael, Benjamin Harrison, Kristy L. Townsend

**Affiliations:** 1 Graduate School of Biomedical Science and Engineering, University of Maine, Orono ME, United States of America; 2 School of Biology and Ecology, University of Maine, Orono ME, United States of America; 3 Maine Medical Center Research Institute, Scarborough ME, United States of America; 4 University of New England, Biddeford ME, United States of America; University of Minnesota, UNITED STATES

## Abstract

The difficulty in obtaining as well as maintaining weight loss, together with the impairment of metabolic control in conditions like diabetes and cardiovascular disease, may represent pathological situations of inadequate neural communication between the brain and peripheral organs and tissues. Innervation of adipose tissues by peripheral nerves provides a means of communication between the master metabolic regulator in the brain (chiefly the hypothalamus), and energy-expending and energy-storing cells in the body (primarily adipocytes). Although chemical and surgical denervation studies have clearly demonstrated how crucial adipose tissue neural innervation is for maintaining proper metabolic health, we have uncovered that adipose tissue becomes neuropathic (ie: reduction in neurites) in various conditions of metabolic dysregulation. Here, utilizing both human and mouse adipose tissues, we present evidence of adipose tissue neuropathy, or loss of proper innervation, under pathophysiological conditions such as obesity, diabetes, and aging, all of which are concomitant with insult to the adipose organ as well as metabolic dysfunction. Neuropathy is indicated by loss of nerve fiber protein expression, reduction in synaptic markers, and lower neurotrophic factor expression in adipose tissue. Aging-related adipose neuropathy particularly results in loss of innervation around the tissue vasculature, which cannot be reversed by exercise. Together with indications of neuropathy in muscle and bone, these findings underscore that peripheral neuropathy is not restricted to classic tissues like the skin of distal extremities, and that loss of innervation to adipose may trigger or exacerbate metabolic diseases. In addition, we have demonstrated stimulation of adipose tissue neural plasticity with cold exposure, which may ameliorate adipose neuropathy and be a potential therapeutic option to re-innervate adipose and restore metabolic health.

## Introduction

Since body weight regulation involves a precise balance between energy intake and energy expenditure, and requires coordination and control stemming from the central nervous system (CNS), the brain needs to adequately communicate with peripheral organs and tissues via peripheral nerves in order to maintain metabolic health. The CNS is able to control energy expenditure by signaling motivation to exercise or seek certain foods, by driving sympathetic nervous system (SNS) activation of energy expending processes in the peripheral tissues of the body, and through receiving feedback from tissue sensory nerves, like those from adipose [1]. Sensory nerves in white adipose tissue (WAT) are thought to communicate the status of energy stores to the brain, in order to help regulate energy intake versus expenditure. In WAT, SNS activation serves to increase lipolysis, reduce *de novo* adipogenesis, and stimulate a process termed ‘browning’, whereby uncoupling protein 1 (UCP1)-positive brown adipocytes appear in WAT depots and contribute to energy expenditure via thermogenesis [2]. In brown adipose tissue (BAT), SNS activation also drives non-shivering thermogenesis via activation of mitochondrial UCP1, which is a brown adipocyte-specific gene that is required for this energy expending process in both classical and inducible/recruitable brown adipocytes [3,4]. During thermogenesis, BAT must utilize fatty acid fuels, which are both stored in the multilocular lipid droplets of brown adipocytes and are also obtained via circulating lipids that are released from WAT through lipolysis, a sympathetic-mediated process [5,6].

Surgical and chemical denervation studies have demonstrated the importance of adipose tissue nerves, and denervation leads to a loss of proper metabolic control [3,7,8]. Denervation of WAT also leads to an increase in tissue mass and adipocyte cell number [9–11]. Therefore, the regulation of lipid stores in both BAT and WAT and the activation of energy expenditure through CNS-SNS communication are essential for proper body weight maintenance and metabolic health. It is not yet understood what other aspects of adipose tissue function are under neural control, and whether or not the nerves that innervate adipose tissue can modify their connections or tissue neurite density under physiological or pathophysiological conditions. Therefore, these questions regarding adipose nerve remodeling warrant further investigation.

The nerves that innervate adipose tissues include numerous peripheral nerve subtypes, such as sensory, parasympathetic and sympathetic nerves [12–16]. Although the number of studies assessing adipose innervation are increasing, further demonstrating the importance of brain-adipose communication [17–19], it is still unclear which neurotransmitters and neuropeptides (aside from the well-studied norepinephrine) are released in adipose tissue and onto which receptor-expressing cell types. A better understanding of how the peripheral nerves in adipose tissue are regulated is important for the field, including differences in nerve plasticity between innervation of BAT and WAT, as well as sex differences in neurite density.

In other tissues, and in many organisms, peripheral nerves are appreciated as plastic (ie: able to undergo remodeling of neurites and synapses in response to stimuli), or neuropathic (dying-back in pathophysiological conditions). For example, with distal peripheral neuropathy, nerves in the skin of distal extremities can die back through an unclear process, resulting in pain, loss of sensation, and severe discomfort. The process begins in the skin and moves inward. Neuropathy can be caused by aging, certain drugs (such as antibiotics and chemotherapy agents), or diabetes [20–22]. Diabetic neuropathy is especially prominent, affecting over 50% of diabetic individuals, and often leading to limb amputation [23, 24]. Aging is associated with a loss of metabolic regulation and an increased propensity for diabetes [25–27], and is independently associated with an idiopathic form of peripheral neuropathy [28]. The debilitating aspects of peripheral neuropathy are largely due to the inability to prevent or treat these conditions, and the inability to halt and reverse the neurodegeneration. Standard clinical approaches include pain management or glucose regulation (with diabetic neuropathy), but no therapies are currently approved to mitigate nerve die-back or to stimulate peripheral nerve re-growth or re-myelination. It is important to understand whether or not neuropathy can extend below the skin into underlying adipose tissue, which may promote or exacerbate metabolic disease.

Given the close association among situations of metabolic dysregulation (aging, obesity, diabetes) and peripheral neuropathy, and the clear importance of adipose tissue innervation for metabolic homeostasis, we sought to determine if adipose tissue nerves also undergo neuropathy with these conditions. To do this, we assessed human adipose tissue samples across a range of ages and body mass indices (BMIs), and also utilized mouse models of aging and obesity/diabetes. We hypothesized that age and obesity/diabetes would be positively correlated with a loss of proper innervation of adipose tissues. We also sought to determine which interventions could stimulate re-innervation of adipose tissue in situations of adipose neuropathy, through a beneficial and physiological process of nerve plasticity.

## Methods

### Mice, metabolic phenotyping, and in vivo analyses

#### Young/aged and sedentary/exercised mice

Age-matched C57BL/6J male mice (Jackson Laboratory, Bar Harbor, ME; stock number *000664 Black 6*) were mated and aged to 16 months at University of Maine. Body weight and adiposity were compared to age-matched C57BL/6J male mice at 10–12 weeks old.

#### Young sedentary/exercised mice

Young (12–15 week old) C57BL/6J male mice (Jackson Laboratory, Bar Harbor, ME; stock number *000664 Black 6*) were imported and then age and body weight matched before randomized assignation to either sedentary or exercised groups.

#### Voluntary running-wheel exercise

Young and aged animals were single-housed in running wheel cages that allowed *ad libitum* access to running, for a period of 7 days. Control (sedentary) animals were either single-caged with locked running wheels or caged in pairs without a running wheel.

#### BTBR ob/ob

Male BTBR +/+ (WT) and *ob*/*ob* mutant (MUT) mice (Jackson Laboratory, Bar Harbor, ME; BTBR.Cg-*Lep*^*ob*^/WiscJ, stock number 004824) were fed a standard chow diet and aged to a minimum of 12 weeks of age, when they exhibit a robust phenotype: including obesity, diabetes, and hyperglycemia [29]. These animals (aged 12–28 weeks) were then euthanized after Von Frey Analysis of tactile allodynia.

#### Von frey analysis

A Von Frey mechanical nociceptive assay was performed on BTBR mice ranging from 12–24 weeks of age, to determine tactile sensitivity of hind paw skin, according to protocol described by [30]. Briefly, each mouse was subjected to five filaments (*Semmes-Weinstein* evaluators) at varying strengths (4.56, 4.31, 4.08, 3.61, 2.36), which corresponded to specific target forces (4, 2, 1, 0.4, 0.02 –grams of force (g), (Stoelting Co., Wood Dale, IL)). For the Von Frey evaluations, mice were placed on top of a grid platform into individual clear-walled compartments and allowed to acclimate with no stimulus for at least 20 minutes. After the acclimation period, trials began and filaments were applied in order of decreasing strength. Each filament was applied to the mid-plantar surface of the hind paw and slight pressure applied until the mouse showed a response or the filament bent with force. Mouse response was recorded as either positive (immediate paw removal or paw licking when filament is applied), neutral (delayed paw removal), or negative (no reaction), and each filament strength test was performed in 5 cycles. For tissue harvesting all animals were CO_2_ euthanized followed by cervical dislocation.

#### CL316,243 injections

Adult (12–13 week old) male C57BL/6 mice received daily i.p. injections of ADRβ3 agonist CL316,243 (Tocris Bioscience, Bristol, U.K.; Cat # 1499), at 1.0 mg/kg BW or an equivalent amount of sterile saline, for 10–14 days.

### Collection of adipose secretions and BDNF ELISA

Inguinal scWAT depots were dissected, weighed and minced in a petri dish containing DMEM (high-glucose, serum-free). Minced tissue was transferred to a 15mL conical tube, with 5mL DMEM (loosely capped to keep tissue oxygenated) and placed in a shaking water bath at 37°C. Secretions were collected at time 0, 1hr, 2hrs, and 3hrs (1mL collected from conical tube at each time point and replaced with 1mL fresh DMEM). Secretions were stored at -80°C until processing. For ELISA, protein secretions were concentrated using Amicon^®^ Ultra Centrifugal Filters, Ultracel^®^-100K (Millipore, Burlington, MA USA; Cat. # UFC510096), per manufacturer’s instructions. Mouse BDNF PicoKine™ ELISA Kit (Boster Biological Technology, Pleasanton, CA, USA; Cat# EK0309) was used per manufacturer’s instruction to determine amount of BDNF present in adipose active secretions.

### Human adipose tissue analyses

Human adipose samples (subcutaneous and omental) were obtained from the Boston Nutrition Obesity Research Center (BNORC) adipose tissue core. Biopsies of scWAT and omental adipose were taken from patients during elective surgery. All patients were either non-diabetic or pre-diabetic, with the exception of four diabetic individuals, 2 of whom were in the BMI cohort and 2 in the aged cohort (indicated by asterisks in data plots; see S1–S3 Tables for relevant patient data). Frozen and fixed tissue (from only a subset of patients) samples were obtained for analyses, including histology and western blotting.

### Mouse adipose tissue collection and analyses; immunostaining

Mice were euthanized by CO2 asphyxiation with secondary cervical dislocation, whole subcutaneous white adipose tissue (scWAT) depots were carefully removed to remain intact, and fixed in 2% PFA at 4°C for 4hr-12hrs depending on thickness of tissue. The tissues were then rinsed for 10 minutes with 1X PBS w/ 10u/mL heparin, twice at 4°C. Tissues were incubated in blocking buffer (1XPBS/2.5% BSA/0.5–1% Triton) at 4°C at least overnight but no more than 7 days, depending on tissue size, with blocking buffer replaced every 24hr period. After blocking period, tissues were flattened by being placed between two large glass slides bound tightly together, for at least 30min but no more than 1.5hrs at 4°C. Tissues were next incubated with 0.1% Typogen Black for 20 minutes at room temperature on a rotating platform to minimize autofluorescence. Following Typogen Black incubation, tissues were washed with 1X PBS w/ 10u/mL heparin on rotating platform at 4°C replacing PBS every 1hr for a total of 4-6hrs, or until all unbound stain was removed. Immunostaining of innervation with primary antibodies was performed overnight at 4°C, and the following day tissues were washed with 1XPBS on a rotating platform at 4°C, replacing PBS every 1hr for a total of 4-6hrs followed by incubation with secondary fluorescent antibodies. Primary antibodies included: PGP9.5 (1:1000, Abcam, Cambridge, U.K. Cat. #10404 and #108986); beta-3 tubulin, (6G7, 1:250) from Developmental Studies Hybridoma Bank, (University of Iowa, USA). Secondary antibodies included Goat anti-Rabbit IgG H+L Alexa Fluor 488 at 1:500 from Molecular Probes. For vascular autofluorescence visualization tissues were not washed in 1X PBS w/ 10u/mL heparin, prior to further immunostaining. For vascular staining, tissues were incubated with isolectin IB_4_ stain conjugated to Alexa 594 (ThermoFisher Scientific, Waltham, MA, USA; Cat # I21413) at 1μg/mL concentration overnight at room temperature. Tissues were washed in 1X PBS and mounted on slides. Images were acquired using Nikon Eclipse E400 epiflourescent microscope (Nikon, Minato, Tokyo, Japan) or Leica TCS SP8 (Leica Microsystems, Wetzler, Germany) digital lightsheet/confocal microscope.

### Whole depot imaging and analysis

Mouse inguinal subcutaneous adipose depots were removed intact and immunostained as described above. Entire depots were imaged with a 10x objective on a Leica TCS SP8 or DMI6000 confocal microscope (Leica Microsystems, Wetzlar, Germany), by tiling z-stacks of the entire depth of tissue. Images ranged between 542–3464 tiles per depot, the average lied at approximately 1000 tiles per depot. Tiles were individually Z-projected and background subtracted (using the rolling ball method). Processed tiles were then thresholded into binary images and skeletonized. To analyze arborization of adipose nerves, skeletons were assessed for the following parameters: innervation density, total number of branches, total skeleton length and tortuosity. Branches less than 4μm in length were excluded from the analysis. All analyses were performed in FIJI image analysis software. Arborization parameters were normalized to tissue weight.

### Neuromuscular junction immunofluorescence, imaging, and analysis

Following protocols provided by Greg Cox and Robert Burgess at Jackson Laboratory, mice were euthanized, both soleus and medial gastrocnemius tissues were carefully removed and fixed in a 2% PFA at 4°C for 2 hours. The tissues were then gently rinsed with 1XPBS and incubated in blocking buffer (1XPBS/2.5%BSA/0.5–1%Triton) at 4°C for at least 24 hours but no more than 7 days. After blocking period muscles were teased, tendons and fat were removed and muscle tissue was flattened by being placed between two tightly-bound glass slides for at least 30 minutes at 4°C. Tissues were next transferred to fresh blocking buffer at 4°C for at least 12 hours, but no more than 7 days. Immunostaining of innervation with primary antibodies was performed overnight at 4°C, the following day tissues were washed with 1XPBS on a rotating platform at 4°C replacing PBS every 1hr for a total of 4-6hrs. Tissues were then incubated with fluorescent secondary antibodies overnight and washed again in 1XPBS on a rotating platform at 4°C replacing PBS every 1hr for a total of 4-6hrs. Primary antibodies included: neurofilament-M (2H3, 1:500) and synaptic vesicles (SV2, 1:250) from Developmental Studies Hybridoma Bank, (University of Iowa, USA). Secondary antibodies included: Alexa Fluor 488 at 1:500 (A21121) and alpha-bungarotoxin (BTX)-conjugated to Alexa Fluor 594 at 1:1000 (B13423) from Molecular Probes (Eugene, OR, USA). Tissues were next mounted on microscope slides using Millipore mounting fluid (Burlington, MA USA; Cat. # 5013) and 1 1/5 coverslips then sealed and allowed to set overnight. All images were acquired with Nikon Eclipse E400 epiflourescent microscope using an integrated ‘Real Time Manual EDF’ acquisition tool in Nikon Elements Software (Nikon, Minato, Tokyo, Japan). Insert images were cropped in FIJI. Brightness, contrast, and sharpness were adjusted in Microsoft PowerPoint. Up to 100 NMJs were counted for each tissue and statistics were conducted in GraphPad PRISM software (La Jolla, CA, USA) using the multiple t-tests (one-per row) function.

### BTBR bone analysis

Femurs were harvested from 12–13 weeks old BTBR WT and MUT male mice, N = 5 for WT, N = 4 for MUT. Micro-architecture of the distal trabecular bone and midshaft cortical bone of the femur was analyzed by μCT (resolution 10 μm, VivaCT-40, Scanco Medical AG). Measurements included femur bone volume/total volume (BV/TV) and trabecular bone mineral density (tb. BMD). Cortical region scans were measured at the midpoint of each femur, with an isotropic pixel size of 21 μm and slice thickness of 21 μm. This allowed calculation of cortical thickness (Ct.Th.). All scans were analyzed using manufacturer’s software (Scanco Medical AG, version 4.05).

### RNA extraction and gene expression

Total RNA was isolated from tissues using a Trizol reagent (Zymo, Irvine, CA, USA; Cat. # R2050-1-200), Bullet Blender for lysis, and Zymo Miniprep kit (Zymo, Irvine, CA, USA; Cat. # R2052). RNA yield was determined on a Nanodrop and cDNA synthesized using High Capacity Synthesis Kit (Applied Biosystems, Foster City, CA, USA; Cat# 4368813). Real-time quantitative (q)PCR was performed with SYBR Green (Bio-Rad) and CFX96 or CFX384 real-time PCR detection system (Bio-Rad, Hercules, CA, USA). PrimePCR™ Probe Assays were used for the following genes: *Bdnf*, *Ngf*, and *Ntf3* (Bio-Rad; Assay ID# qMmuCEP0058759, qMmuCIP0042317, qMmuCEP0042141, respectively). Relative quantification analysis and fold change was performed with all values normalized to levels of a housekeeper gene (cyclophilin). Primer sequences are listed in S5 Table.

### Western blotting

Protein expression was measured by western blotting analysis of tissue lysates. Whole adipose depots were homogenized in RIPA buffer with protease inhibitors in a Bullet Blender, followed by Bradford Assay, and preparation of equal-concentration lysates in Laemmli buffer. 60ug was loaded per lane of a 10% polyacrylamide gel, and following gel running proteins were transferred to PVDF membranes for antibody incubations. Anti-GAP43 (Novus Biologicals, Cat# NB300-143) at 1:10,000 dilution was used with mouse protein lysates. Anti-Tyrosine hydroxylase (TH) (Millipore Cat. # AB152) at 1:1000 dilution was used with both mouse and human protein lysates. Anti-PGP9.5 antibody (Abcam Cat. #ab10404 and #ab108986) was used at a 1:1000 and 1:500 dilutions respectively, were used with both mouse and human protein lysates. Anti-PSD95 antibody (Abcam Cat. #ab18258) at 1:750 dilution was used with both mouse and human protein lysates. GAP43, TH, PGP9.5, and PSD95 protein expression was normalized to one of the following housekeeping proteins, all at 1:1000 dilutions: β-Tubulin (Cell Signaling Technology, Danvers, MA, USA; Cat. # 2146BC), β-Actin (Abcam, Cambridge, U.K.; Cat. # ab8227), Cyclophilin B (Abcam, Cambridge, U.K.; Cat. #ab16045). Secondary antibody anti-rabbit HRP-linked (Cell Signaling Technology, Danvers, MA, USA; Cat. # 7074) at 1:3000 dilution was used for conjugation with all primaries. Blots were visualized on a Syngene G:BOX (Frederick, MD, USA). Ponceau S staining was performed after immunoblotting was complete using Ponceau S Solution (Sigma-Aldrich, St. Louis, MO, USA; Cat. # P7170-1L).

### Cold exposure experiments

All cold exposure was carried out in a diurnal incubator (Caron, Marietta, OH, USA) at 5°C with physiological humidity and a 12hr light cycle. Animals were housed two to a cage and continuously cold exposed for 3–14 days.

For whole adipose innervation imaging, 18–22 week old control male mice on a mixed genetic background were housed either at room temperature, cold exposed for 10 days, or cold exposed for 10 days and returned to room temperature for 1 week (‘rewarmed’).

### Statistical analysis

For all animal experiments, mice were randomized to treatment groups to ensure no difference in starting body weight. All plots represent mean +/-SEM. Statistical calculations were carried out in Excel or GraphPad Prism software (La Jolla, CA, USA), utilizing ANOVA, Linear Regression, or Student’s t-test as indications of significance (specified in Figure legends). Gene and protein expression data were normalized to a housekeeper and analyzed by either ANOVA or by Student’s t-test, two-tailed, using Welch’s correction when variance was unequal. Error bars are SEMs. For all figures, *p < 0.05, **p < 0.01, ***p < 0.001, ****p < 0.0001.

### Ethical statement

All procedures and handling of animals were performed in accordance with the University of Maine’s Institutional Animal Care and Use Committee (IACUC), to comply with the guidelines of the PHS Policy on Humane Care and Use of Laboratory Animals, and Guide for the Care and Use of Laboratory Animals. This study was approved by the University of Maine’s IACUC, under protocol A2017-09-04. Human tissue samples were obtained from the Boston Nutrition and Obesity Research Center (BNORC) and were de-identified prior to being provided to our laboratory. Tissues were collected by BNORC under their IRB-approved protocol.

## Results

### Obese and diabetic mice display subcutaneous white adipose tissue (scWAT) neuropathy

In order to investigate whether obesity/diabetes leads to adipose tissue neuropathy, we used BTBR mice with the *ob/ob* leptin-deficient mutation (MUT). These animals develop severe obesity, type 2 diabetes, hypercholersterolemia, and insulin resistance, with significantly increased body weight and blood glucose by 8 weeks of age [29]. Although disease progression is slower in females when compared to males [31], both sexes eventually develop diabetes and peripheral neuropathy, with nerve conductance deficits and intraepidermal nerve fiber loss evident by 9 and 13 weeks, respectively [29]. While this diabetic neuropathy has been well described in skin and paw, the effects on adipose innervation, as well as innervation of other tissues, have not been assessed.

**Fig 1:**
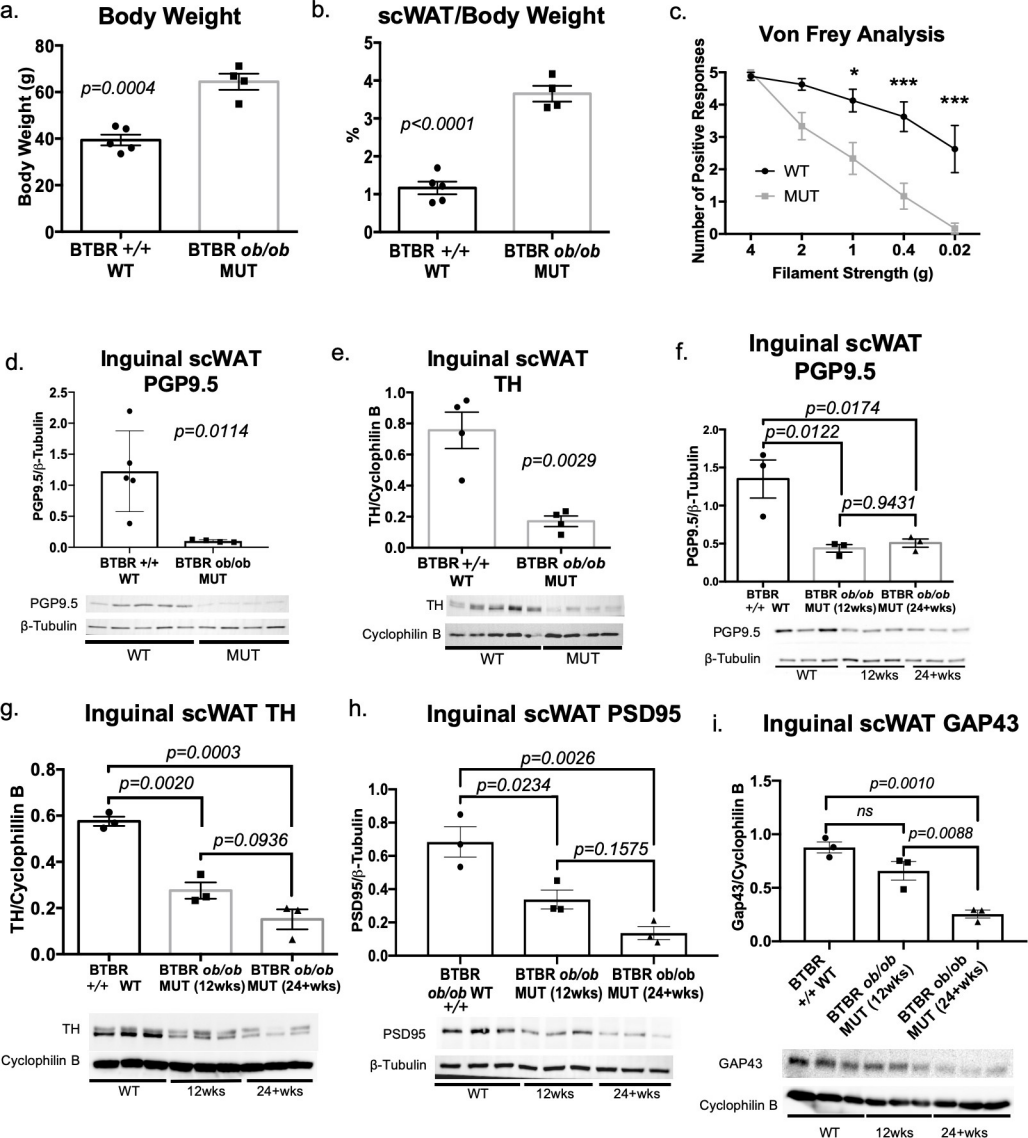
Obesity and diabetes led to white adipose tissue neuropathy. The BTBR *ob/ob* (MUT) model of obesity and diabetes was compared to BTBR +/+ wild-type (WT) for body weight measurements (a), and adiposity (b). Body and tissue weight data were analyzed by two-tailed Student’s t-test. Von Frey tactile allodynia analysis was performed on MUT and WT animals to determine onset of peripheral neuropathy (c). Von Frey data was analyzed by ANOVA with Sidak’s *post hoc* test. For (a-c), all males; WT N = 8, 12–20 weeks old; MUT N = 6, 12–24 weeks old. Protein levels of PGP9.5 (d), as well as TH (e) in inguinal scWAT of the MUT and WT mice were measured by western blotting. For (e), lane 5 was excluded from analyses due to uneven resolution of housekeeper. For (d-e), all males; WT N = 5, 12–20 weeks old and MUT N = 4, 12–20 weeks old. Protein expression of PGP9.5 (f), TH (g), PSD95 (h), and GAP43 (i) in inguinal scWAT of 12–25 weeks old WT, 12 week old MUT and 24–28 week old MUT was measured by western blotting. All males, N = 3 per group. Western blot data were normalized to either β-Tubulin or Cyclophilin B, band intensity were quantified in Image J, and analyzed by two-tailed Student’s t-test. Error bars are SEMs.

By 12 weeks of age, male BTBR MUT mice exhibited the expected increase in body weight when compared to BTBR +/+ wild type (WT) mice (Fig 1A), which was concurrent with increased subcutaneous adiposity (Fig 1B). In a pilot cohort that also included heterozygous (+/- HET) BTBR mice, only MUT animals displayed a trend for increased body weight and inguinal scWAT weight, for both males and females (S1A Fig). Thus, HET mice were not included in subsequent analyses. Consistent with previous reporting, neuropathy was detectable in BTBR MUT mice around 12 weeks of age via a Von Frey mechanical nociceptive assay (male mice, Fig 1C; female mice, S1B Fig). This test determines tactile sensitivity in the skin of the hind paw, as an indirect measure of small fiber peripheral neuropathy. These data revealed that male mice displayed a stronger phenotype of peripheral neuropathy of extremities (Fig 1C compared to S1B Fig), consistent with previous reports [31]. Of note, a reduction in sensitivity is observed late in neuropathy (as seen here), while a hypersensitivity is observed at earlier time-points, which we did not assess.

The pan-neuronal marker protein gene product 9.5 (PGP9.5, also known as ubiquitin C-terminal hydrolase L1 (UCHL-1)), was also greatly reduced in inguinal scWAT of MUT mice (Fig 1D; S1C Fig), indicating a loss of total nerve supply. BTBR MUT mice also displayed a marked reduction in sympathetic activation/innervation in inguinal scWAT as evidenced by a substantial decrease in tyrosine hydroxylase (TH) protein levels (Fig 1E, S1D Fig). TH is the rate-limiting enzyme in catecholamine biosynthesis, including norepinephrine, the main sympathetic nerve neurotransmitter in WAT, and an indicator of SNS activation and innervation. After 12 weeks of age, the loss in total inguinal scWAT innervation remained consistent in MUT mice (Fig 1F), but TH showed a trend for further decrease in MUT mice aged to 24 weeks (Fig 1G). As loss of synaptic connections is another important factor in peripheral neuropathy, we measured protein expression of the synaptic protein postsynaptic density 95 (PSD95), and observed a reduction in PSD95 in 12-week-old MUT mice when compared to WT (Fig 1H). PSD95 protein levels were also further decreased in 24-week-old MUT animals, although this did not reach significance (Fig 1H). Growth-associated protein 43 (GAP43; also known as neuromodulin) is involved in axonal growth cone formation and presynaptic vesicle function, and was decreased in 24-week-old MUT animals (Fig 1I). Together, a reduction in adipose innervation, likely representing adipose neuropathy, was observed in this obese/diabetic model.

**Fig 2:**
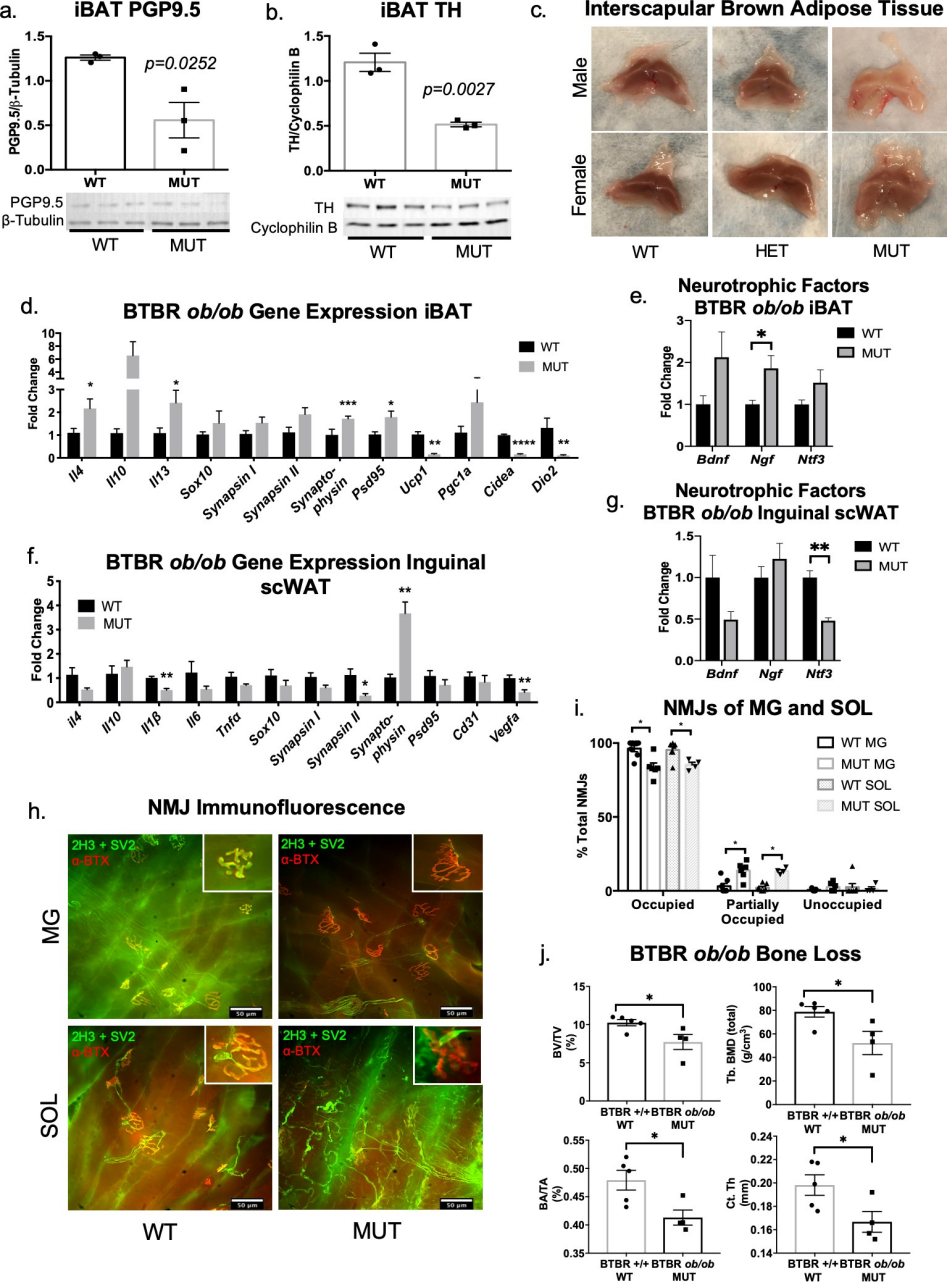
Neuropathy extends beyond white adipose tissue in BTBR *ob/ob* mice. Protein expression of PGP9.5 (a) and TH (b) in iBAT was measured in MUT mice and compared to WT littermate controls; for (a-b) all 12 week old males, N = 3 WT/MUT. For all western blots, data were normalized to housekeeper proteins β-Tubulin or Cyclophilin B, band intensities were quantified in Image J, and analyzed using a two-tailed Student’s t-test. Error bars are SEMs. Whole BAT depots were compared between female and male BTBR HET, MUT, and WT mice (c). Images are representative, males: MUT N = 2; WT N = 3; HET N = 3; females: MUT N = 1; WT N = 3; HET N = 3; all mice were 12–24 weeks old. Gene expression analysis of BAT (d-e) and inguinal scWAT (f-g). Gene expression data were analyzed by two-tailed Student’s t-test, using Welch’s correction when variance was unequal, N = 4–5 per group. Error bars are SEMs. Neuromuscular Junction (NMJ) Analysis (h-i). Immunofluorescent staining of male BTBR WT and MUT neuromuscular junctions of the medial gastrocnemius (MG) and soleus (SOL) muscles was performed using neurofilament M (2H3) and synaptic vesicles (SV2) (in green) to visualize the pre-synaptic area, and α-bungarotoxin (in red) to visualize the post-synaptic area. Representative images at 40x magnification of BTBR *ob/ob* wild-type (WT) medial gastrocnemius (MG) (top left panel), and soleus (bottom left panel) captured on Nikon Eclipse E400 microscope. Mutant (MUT) MG (top right panel) and SOL (bottom right panel) (h). Inserts are of occupied (left panels) and partially occupied (right panels) NMJs in representative images (h). Percent of total NMJs for WT and MUT animals in both MG and SOL muscles was calculated as an indicator of neuropathic state (i). Analysis shows multiple t-tests (per row) of replicate cohorts; N = 7 for WT MG, N = 6 for MUT MG, N = 7 for WT SOL, and N = 4 for MUT SOL. Micro CT analysis of BTBR WT and MUT femurs (j). Bone volume density shown as fraction of bone volume/total volume (BV/TV, top left panel); total bone mineral density (BMD) for trabecular bone (Tb) in top right panel; bone area/total area (BA/TA) bottom left panel; and cortical (Ct) thickness (Th) in bottom right panel (j). All animals were male, 12–13 weeks old, N = 5 for WT, N = 4 for MUT. Data analyzed by two-tailed Student’s T- test. Error bars are SEMs. *p < 0.05, **p < 0.01, ***p < 0.001, ****p < 0.0001.

Because the energy expending intrascapular brown adipose tissue (iBAT) is known to be highly innervated [32, 33], we examined iBAT innervation in the BTBR mice. Protein expression of PGP9.5 revealed a significant decrease in total innervation of iBAT in BTBR MUT animals when compared to littermate controls (Fig 2A). These findings were concurrent with a decrease in protein expression of TH in the iBAT of BTBR MUT mice, indicating decreased sympathetic activation/innervation (Fig 2B). iBAT gross morphology also revealed a marked increase in lipid accumulation, also called “whitening”, in both male and female mutant BTBR mice, but most prominently in male mice that exhibited the stronger neuropathy phenotype at this age (Fig 2C). For BTBR MUT mice, iBAT gene expression showed a significant decrease in *Ucp1*, whose activation by the SNS is necessary for thermogenesis to occur, along with reductions in the brown adipocyte markers *Cidea* and *Dio2*, despite no decrease in synaptic markers (*Synapsin I*, *Synapsin II*, *Synaptophysin*, *Psd95*), or other indicators of nerve health such as the Schwann cell marker *Sox10* (Fig 2D) or neurotrophic factors (Fig 2E). Instead, synaptic markers (*Synaptophysin*, *Psd95)* (Fig 2D) and nerve growth factor (*Ngf)* (Fig 2E), showed increased expression in iBAT of MUT mice, perhaps indicating a compensatory mechanism. The gene expression pattern in inguinal scWAT of BTBR MUT was opposite of what we observed in iBAT and included a coordinated trend for reduced expression of neural markers in MUT animals (Fig 2F and 2G). Together, these data indicate depot-specific differences related to innervation with obesity/diabetes and suggest that different mechanisms may be involved in maintaining nerve health in different adipose depots.

To determine whether peripheral neuropathy extended beyond adipose in BTBR MUT mice, we examined the neuromuscular junctions (NMJ). Immunofluorescent staining of NMJs revealed that BTBR MUT mice had fewer fully occupied NMJs in both the medial gastrocnemius (MG) and soleus (SOL) muscles when compared to WT littermates, accompanied by an increase in partially occupied junctions (Fig 2H and 2I). As there was no difference in unoccupied NMJs, neurodegeneration at the NMJ may be a slower neurodegenerative process with obesity and diabetes than in skin and underlying adipose. This fits with the idea that neuropathy begins in the skin and moves inward.

Bone is highly innervated, and it has been suggested that peripheral neuropathy can affect bone mineral density leading to increased fracture risk [34, 35]. Therefore, using micro-computed tomography (μCT), we assessed trabecular and cortical bone of BTBR MUT animals and compared to WT littermates. We found that bone volume density, trabecular bone mineral density, cortical bone area/total area as well as cortical bone thickness were all reduced in BTBR MUT mice (Fig 2J), potentially due to reduced innervation in that tissue as well.

### White adipose tissue from obese humans exhibits neuropathy

**Fig 3:**
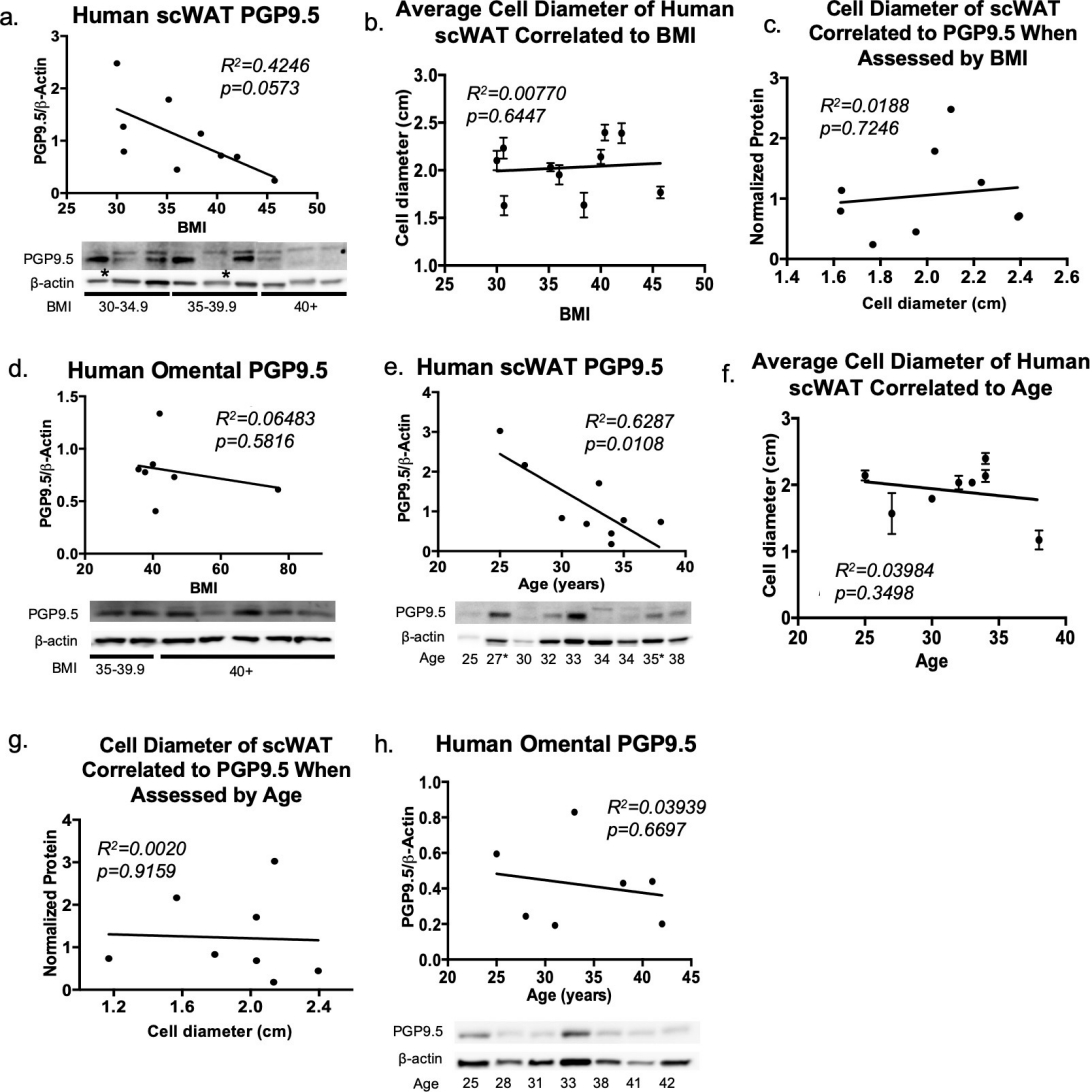
Human subcutaneous and omental white adipose tissue innervation. In human scWAT, protein levels of pan-neuronal marker PGP9.5 were measured by western blotting; linear regression analysis was performed for assessment of normalized protein levels compared to body mass index (BMI) (a). Average cell diameter in scWAT of human samples when assessed by BMI (b), and normalized protein expression plotted against average cell size (c). Protein levels of PGP9.5 were measured in omental adipose by western blotting; linear regression analysis was performed for assessment of normalized protein levels compared to BMI (d). Protein levels of PGP9.5 measured in human scWAT, linear regression analysis was performed for assessment of normalized protein levels compared to age (e). Average cell diameter in scWAT of human samples when assessed by age (f), and normalized protein expression plotted against average cell size (g). Protein levels of PGP9.5 were measured in omental adipose by western blotting; linear regression analysis was performed for assessment of normalized protein levels compared to age (h). Western blot data were normalized to β-actin, band intensities were quantified in Image J, and analyzed by two-tailed Student’s t-test. Protein expression was normalized to β-Actin and band intensities were quantified in Image J. Cell diameter was measured from images of histological cross-sections of adipose tissue from each patient, averaged (n = 3), and analyzed by linear regression. Due to limitations of available samples from the BNORC adipose tissue core at Boston Medical Center, the BMI distribution of omental adipose was clustered around 40. Error bars are SEMs. For (a,e) N = 9 BMI cohort, N = 9 Age cohort. For (a,e) * indicates individuals with diagnosed diabetes. The majority of human samples were females, see S1–S3 Tables for clinical details.

Based on our findings in the BTBR mouse model we sought to determine if neuropathy exists in adipose tissue of obese humans. We investigated degree of innervation in human scWAT and omental WAT obtained from individuals who underwent elective surgery. Protein expression of the pan-neuronal marker PGP9.5 revealed a decreasing trend with increasing BMI in human scWAT adipose tissue (Fig 3A), despite no difference in adipocyte diameter with increasing BMI and no correlation of adipocyte diameter to PGP9.5 levels (Fig 3B and 3C), indicating that adipose neuropathy also likely exists in human tissues. Since we were unable to obtain an entire WAT depot from humans (as we do in mice in order to account for heterogeneity in tissue innervation), and in order to determine if innervation status was confounded by cell size, adipocyte diameter was quantified for the human samples and revealed no differences that may confound these analyses.

We also assessed degree of innervation in human omental adipose tissue and found no significant difference in innervation with increasing BMI (Fig 3D), suggesting that adipose neuropathy may be restricted to scWAT depots as neuropathy extends below the skin. However, due to the limited number of omental adipose samples available, along with the narrow range in BMI, further assessments may be warranted to confirm these findings as omental depots may eventually become neuropathic as well. Nevertheless, these data show that human adipose tissue undergoes neuropathy with obesity (likely the leptin-resistant form) similar to the mouse model (leptin-deficient).

### Aging leads to white adipose tissue neuropathy in humans and mice

Since adiposity increases with age concordant to increased risk of developing type 2 diabetes and aging is associated with an idiopathic form of peripheral neuropathy, we sought to determine if there is also a link between adipose neuropathy and aging. When investigating the relationship between adipose innervation and age in humans, linear regression analysis revealed a significant decrease in protein levels of the pan-neuronal marker PGP9.5 with increasing age in inguinal scWAT (Fig 3E) despite no difference in adipocyte size (Fig 3F), with no correlation between average cell size and protein expression (Fig 3G). Again, this finding did not extend to omental WAT (Fig 3H).

**Fig 4:**
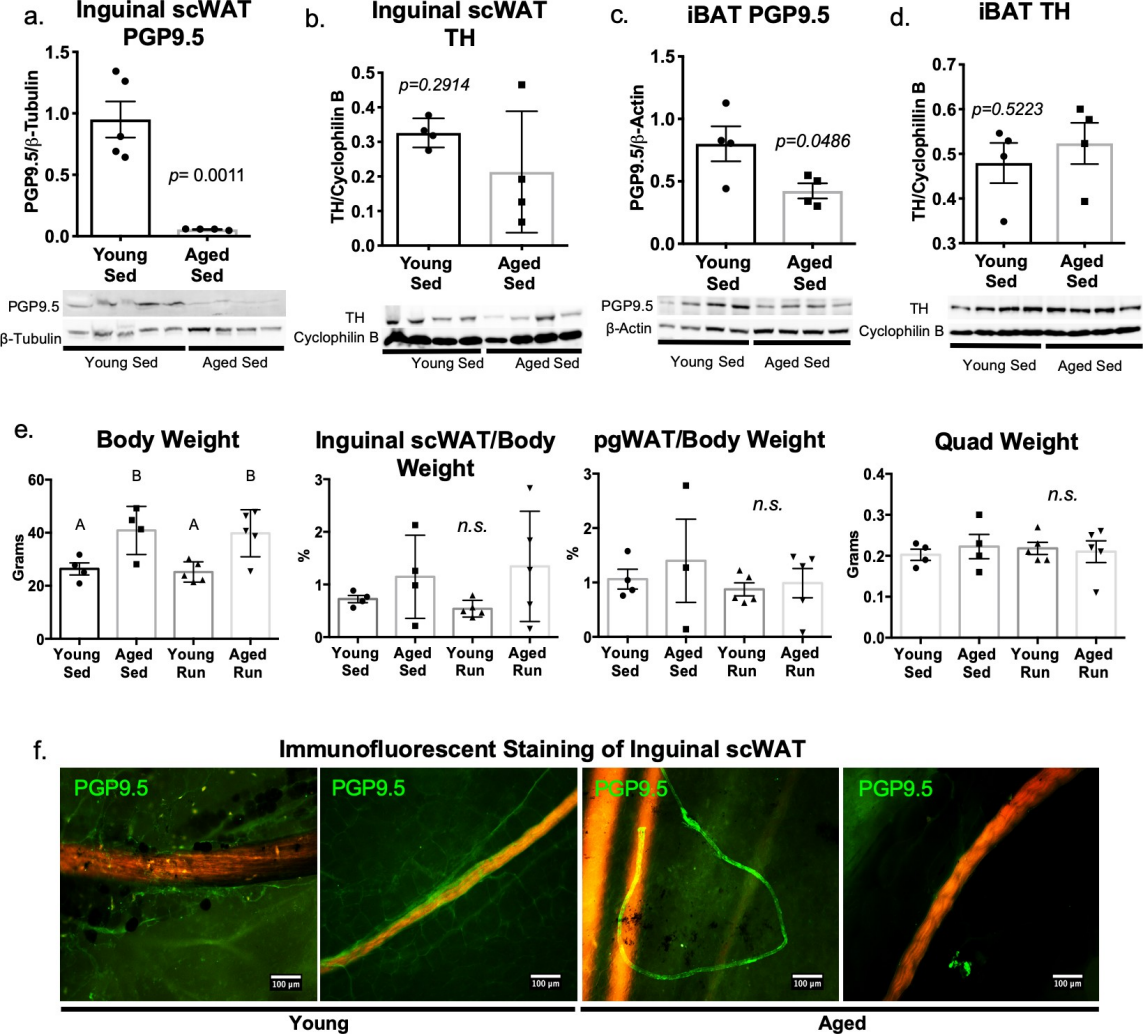
Aging is associated with adipose tissue neuropathy. In young (10–12 weeks old) and aged (16 months old) sedentary male C57BL/6J mice, western blotting was used to measure protein expression of PGP9.5 in inguinal scWAT for assessment of total innervation (a) while TH was used to assess sympathetic activation (b). In the same animals, protein expression of PGP9.5 (c) and TH (d) in intrascapular brown adipose tissue (iBAT) was measured by western blot. Protein expression was normalized to either β-Actin, β-Tubulin or Cycophilin B, band intensity was quantified in Image J and analyzed by two-tailed Student’s t-test; N = 4 for young and aged groups. Body weight, adiposity (inguinal scWAT/body weight & pgWAT/body weight), and quadricep muscle weight was measured for young (12 weeks old) and aged (16 month old) mice under sedentary (sed) and exercised (run) conditions (e). Body and tissue weight analyzed by one-way ANOVA, with Tukey post hoc, groups labeled with the same letter (A or B) are statistically similar, for body weight: *p = 0*.*0473* for young sed v. aged sed; *p = 0*.0240 for young run v aged run. For young and aged sedentary animals N = 4, for young and aged exercised (run) mice, N = 5 per group. Whole depot immunofluorescent imaging of inguinal scWAT from sedentary animals for total innervation (PGP9.5 in green) and vasculature (red/orange; autofluorescence) was performed (f). Images captured at 10x on Nikon Eclipse E400 microscope and are representative of N = 4 mice analyzed per group. Error bars are SEMs, *p < 0.05, **p < 0.01, ***p < 0.001, ****p < 0.0001.

To further explore this relationship, we employed an aged mouse model. Male C57BL/6J mice were aged to 16 months, and the state of their adipose innervation was compared to young mice at 10–12 weeks old. Protein expression of PGP9.5 in inguinal scWAT was decreased in 16 month old aged mice when compared to young mice at 10–12 weeks of age (Fig 4A), with a trend for decreased TH levels (Fig 4B). The same trend for neuropathy with aging, although with less severity, was observed in iBAT of these animals (Fig 4C and 4D). Consistent with the metabolic consequences of middle age, total body mass of aged mice was significantly greater than that of young animals, however, there was no significant difference in adipose depot weight or quadriceps muscle weight, although inguinal scWAT and perigonadal (pg)WAT depot weight did display a trend to be higher at this age (Fig 4E).

We took advantage of vascular autofluorescence [36] to visualize adipose blood vessels in combination with immunofluorescent staining of nerves with a new whole-depot 3D microscopy technique developed in our lab (see Methods section). This imaging revealed that aged mice exhibit a striking loss of innervation around vasculature within their inguinal scWAT (Fig 4F). In young mice, autofluorescent blood vessels (red/orange) are clearly shrouded by fine PGP9.5-expressing nerves (green, Fig 4F, left panels), but by 16 months of age, the adipose vasculature shows a striking loss of this neuronal sheathing (Fig 4F, right panels). There was no change in gene expression of vascular markers, *Cd31* or *Vegfa* between young and aged animals (S2A Fig). Interestingly, there was a trend for decreased gene expression of *Bdnf* and the endothelial derived neurotrophic factor *Ntf3*, although this did not reach significance (S2B Fig). Taken together, these data reveal that loss of proper adipose innervation may precipitate aging-related metabolic dysfunction.

### Exercise and age-related adipose neuropathy

**Fig 5:**
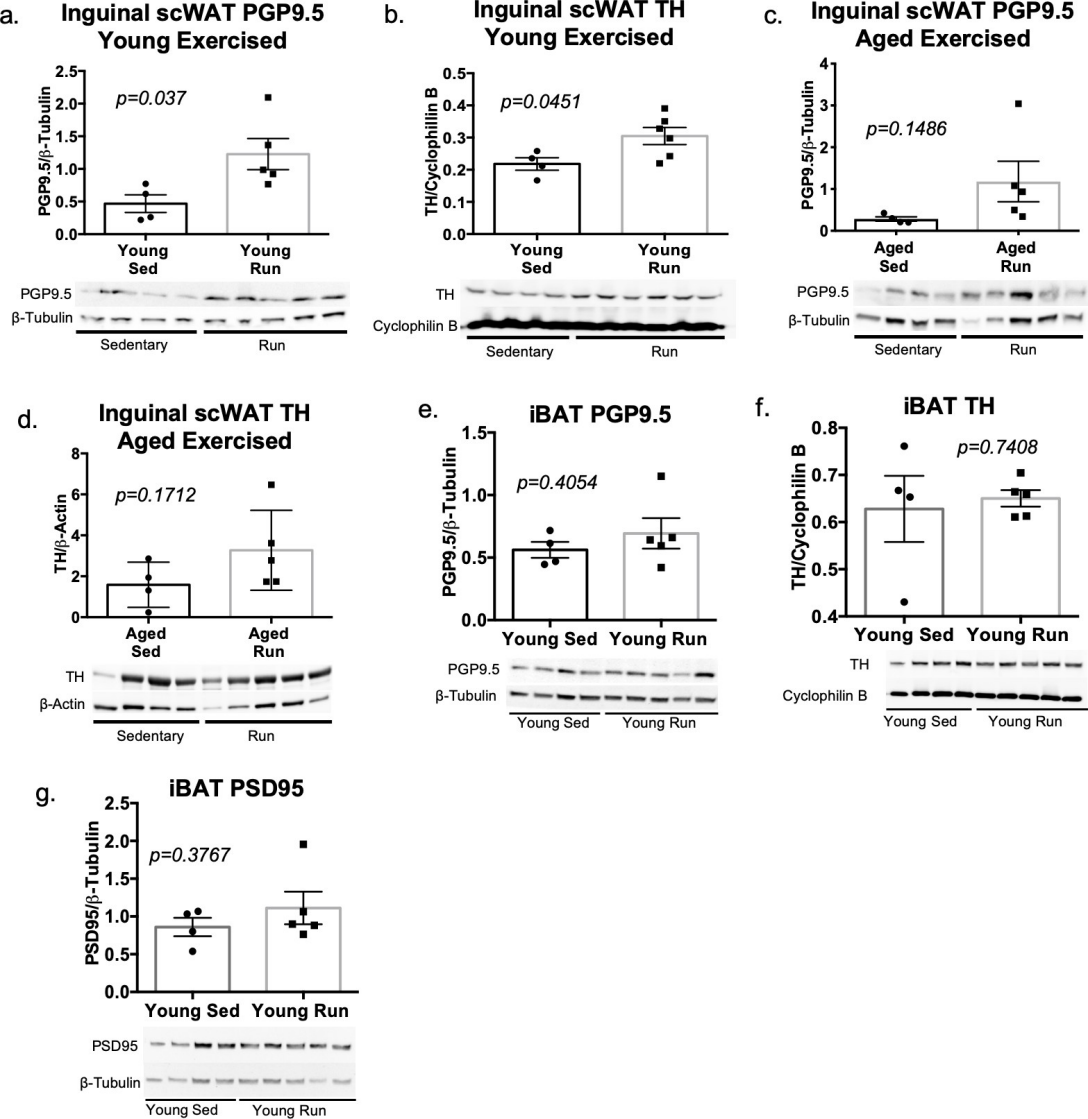
Exercise increases adipose innervation in young mice and attenuated loss of age-related adipose innervation. Young (10–12 weeks old) and aged (16 months old) male C57BL/6J mice were placed in running-wheel cages for 7 days with continuous access to a running wheel (run). To assess exercise effects on adipose innervation in young mice, protein expression in inguinal scWAT was measured by western blotting with PGP9.5 as a marker of total innervation (a), and TH as an indicator of sympathetic activation (b). Protein expression was normalized to either β-Tubulin or Cyclophilin B. Band intensity was quantified in Image J and analyzed by two-tailed Student’s t-test; N = 4 for young sedentary and N = 5–6 for exercised groups. Protein expression of PGP9.5 (c) and TH (d) in inguinal scWAT of aged sedentary and exercised mice was also determined by western blotting. Protein expression was normalized to β-Tubulin or β-Actin, band intensity was quantified in Image J and analyzed by two-tailed Student’s t-test; N = 4 for aged sedentary animals, N = 5 for aged exercised (run) animals. Young (12–15 weeks old) male C57BL/6J mice were placed in running-wheel cages for 7 days with continuous access to a running wheel (run), control (sed) animals were placed in cages with a locked running wheel. Error bars are SEMs. Protein expression of PGP9.5 (e), TH (f), and PSD95 (g) in iBAT of young (12 week old) sedentary (young sed) versus young exercised (young run) mice was determined by western blotting. Protein expression was normalized to β-Tubulin or Cyclophilin B; band density was quantified in Image J and analyzed using a two-tailed Student’s t-test. Error bars are SEMs.

Exercise has been shown to increase circulating levels of the nerve survival factor BDNF and can increase neural plasticity in the brain [37]. We therefore exposed young and aged mice to a short bout of voluntary wheel running for 7 days and assessed the status of their adipose innervation. Protein levels of both PGP9.5 and TH in inguinal scWAT of young exercised mice were significantly increased when compared to young sedentary mice (Fig 5A and 5B), indicating that neurite outgrowth has likely taken place, and that at least a portion of the neurite outgrowth is in sympathetic nerves. Exercise also resulted in a trend for increased protein expression of PGP9.5 and TH in aged animals (Fig 5C and 5D), although this did not reach significance, further supporting a blunted effect with age. The effects of exercise on adipose tissue did not extend to BAT. Protein expression of PGP9.5, TH, and PSD95 in BAT did not differ between young sedentary and exercised groups (Fig 5E–5G).

Exercise did not appear to have a specific effect on vasculature innervation in young or aged animals, but the levels of PGP9.5 around vasculature had high intra-individual variability (S2C Fig). Therefore, to further probe the effects of exercise on vascular innervation, a separate cohort with only young animals was subjected to 7 days of voluntary wheel running as described above. As observed previously (Fig 4E), there was no change observed in body weight or adiposity between young sedentary and exercised animals (S2D Fig). Consistent with other reports [38, 39] there was also no difference in skeletal muscle mass for either sedentary or exercised animals after 1 week of voluntary wheel running (S2D Fig). There was a trend for increased protein expression of the synaptic marker PSD95 in axillary scWAT (S2E Fig). There was an increased trend for Schwann cell marker *Sox10*, neurotrophic factor *Bdnf*, and post-synaptic marker *Psd95*, which did not reach significance (S2F Fig). Innervation of blood vessels was assessed by immunostaining of PGP9.5 in combination with isolectin staining of vasculature in inguinal scWAT. Innervation of blood vessels that measured 50 μm or more in diameter was evaluated (S2G and S2H Fig). We found no difference in either percentage of blood vessels innervated (S2G Fig, left panel) or number of nerves per blood vessel (S2G Fig, right panel) between sedentary and exercised groups, suggesting that exercise is not an effective intervention or that a longer period of exercise is required for tissue re-innervation.

### Assessments of adipose innervation across depot and sex

**Fig 6:**
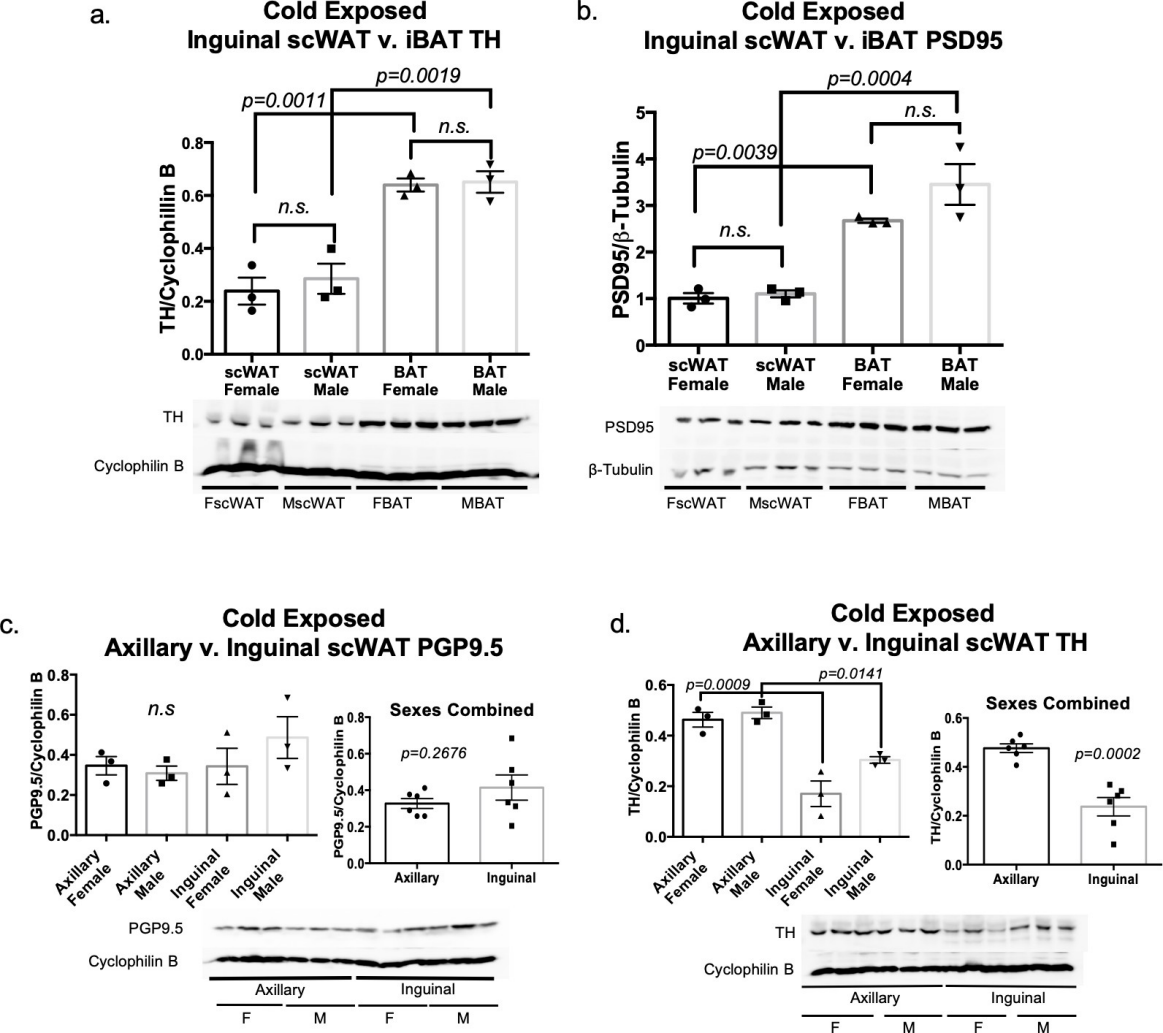
Adipose innervation: Sex and depot comparison. Adult (16 week old) male and female control mice on a C57BL/6J background were cold exposed (5°C) for 3 days. Protein expression of TH (a) and PSD95 (b) were measured in inguinal scWAT and BAT via western blotting. Protein expression of pan-neuronal marker PGP9.5 (c) and sympathetic nerve marker TH (d) was measured in axillary and inguinal scWAT for both sexes via western blotting. Protein expression was normalized to β-Tubulin or Cyclophilin B, band density was quantified in Image J and analyzed using a Two-Tailed Student’s t-test. Error bars are SEMs.

To determine whether a difference in adipose innervation and sympathetic activation exists between depots and/or sexes in healthy animals, 16 week old male and female control mice were cold exposed for 3 days. No difference between sexes was observed for sympathetic innervation/activation (TH) or synapses (PSD95) in inguinal scWAT and iBAT (Fig 6A and 6B), however, both TH and PSD95 protein expression were much greater in iBAT than inguinal scWAT. Similarly, protein expression of PGP9.5 and TH in axillary and inguinal scWAT depots was comparable between sexes (Fig 6C and 6D). However, while PGP9.5 expression remained the same between the depots (Fig 6C), axillary scWAT exhibited significantly more protein expression of TH than inguinal scWAT (Fig 6D), which correlates to the ‘browning’ potential of this particular WAT depot in comparison to inguinal WAT [40].

### Adipose tissue undergoes remodeling with cold exposure

**Fig 7:**
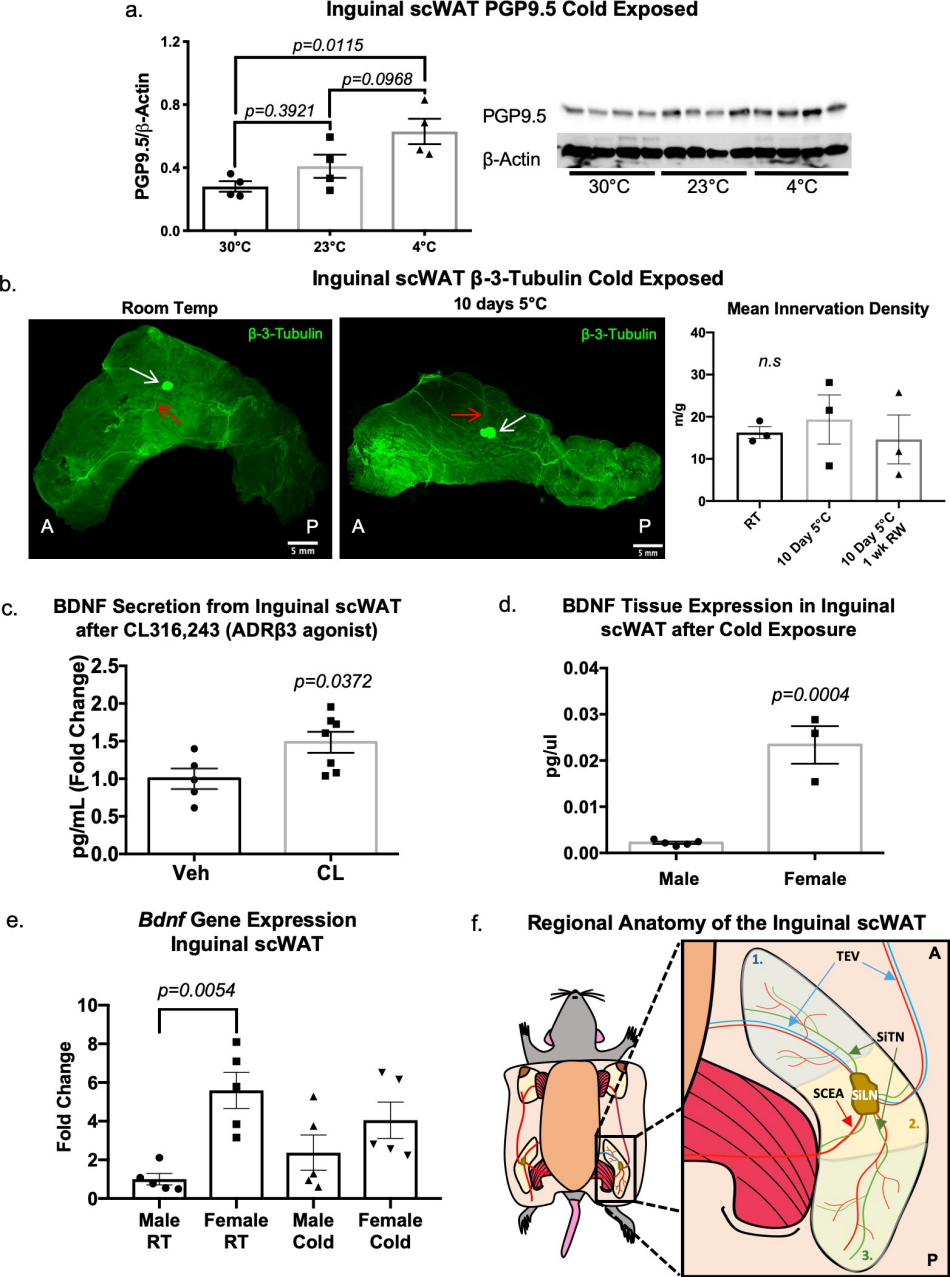
Cold exposure induces adipose nerve remodeling. Adult (8 week old) wild-type C57BL/6J male mice were either cold exposed (at 4°C), maintained at room temperature, or at thermoneutrality (30°C) for 3 days. Changes in innervation were assessed by measuring protein expression of a pan-neuronal marker (PGP9.5) in inguinal scWAT via western blotting (a). Protein expression was normalized to β-Actin, band intensity was quantified in Image J and analyzed by two-tailed Student’s t-test; N = 4 per group. In a separate experiment, adult (18–22 week old) control male mice on a mixed genetic background were maintained either at room temperature (RT), at 5°C for 10 days, or at 5°C for 10 days and then returned to RT for 1 week (rewarmed). Entire depots were immunostained with β3-Tubuilin and imaged on a Leica TCS SP8 or DMI6000 confocal microscope by tiling z-stacks through the entire depth of tissue (b). For quantification of arborization (b, right panel), tiles were individually Z-projected, background subtracted, thresholded into binary images, and skeletonized. Branches less than 4μm in length were excluded from the analysis. White arrows point to subiliac lymph node, red arrows indicate branches of the thoracoepigastric vein (TEV); anterior side (A) is left of subiliac lymph node, posterior side (P) is right of subiliac lymph node. Images are representative of N = 3 per group, error bars are SEMs. Adult (12–13 weeks old) male C57BL/6J mice received either daily i.p. injections of ADRβ3 agonist CL316,243 (at 1.0 mg/kg BW), or vehicle (Veh) for 10–14 days; *ex vivo* secretions (collected at 1hr and 2hr) from inguinal scWAT explants were measured for BDNF by ELISA, analyzed by two-tailed Student’s t-test, and presented as fold change in picograms (pg)/mL (c). Data is representative of multiple cohorts, N = 5 (Veh), N = 7 (CL316,243). Error bars are SEMs. Adult (16 week old) male and female control mice on a C57BL/6J background were cold exposed (5°C) for 3 days. BDNF protein expression in inguinal scWAT was measured for both sexes via multiplex ELISA assay (d). Gene expression analysis of *Bdnf* in inguinal scWAT of 15–17 week old male and female C57BL/6J mice (e). Animals were maintained at room temperature (RT) or cold exposed (5°C) for 7 days; gene expression data were analyzed by two-tailed Student’s t-test, N = 5 per group. Error bars are SEMs. Schematic of inguinal scWAT (f) divided into three anatomically distinct areas: 1. area anterior to subiliac lymph node (LN), 2. LN area, 3. area posterior to LN. Major vasculature is shown with the TEV [65] illustrated as blue line and the superficial caudal epigastric artery (SCEA) [66] and other vasculature illustrated as red lines. Branches of the subiliac transverse nerves (SiTN) are illustrated as green lines.

In addition to exercise as a potential means to promote adipose tissue neural plasticity, cold exposure has been demonstrated to increase TH+ nerve fibers in murine adipose tissue [40, 41]. Similarly, we also found that cold stimulation increased adipose tissue innervation, as protein levels of PGP9.5 went up in scWAT after only 3 days of cold exposure compared to tissues from mice housed at room temperature or thermoneutrality (Fig 7A). To examine the adipose nerve network under this innervation promoting condition, adult mice maintained at room temperature (RT) were compared to littermates that were either cold exposed for 10 days or cold exposed for 10 days and rewarmed for 1 week. Inguinal scWAT depots were immunostained with the pan-neuronal marker β3-Tubulin. At room temperature, a diffuse pattern of innervation can be discerned (Fig 7B, left panel), with larger nerve bundles converging around the subiliac lymph node (white arrow; red arrows point to branches of the thoracoepigastric vein). This staining pattern is similar to the staining pattern we observed with the other pan-neuronal marker, PGP9.5. The posterior end (right of subiliac lymph node in image) of the tissue did not appear to have as much innervation as the anterior portion (left of subiliac lymph node in image) or as the area surrounding the subiliac lymph node.

After cold exposure, a distinct change in the neural arborization pattern was observed (Fig 7B, middle panel), particularly an increase in intensity around the lymph node. Since these 2D representations of 3D data do not convey accurate changes in neurite density, tissue z-stacks were analyzed for mean innervation density (calculated as total axon length normalized to tissue weight) and this revealed a trend for increased innervation density in the 10 day cold exposed group compared to RT and rewarmed groups (Fig 7B, right panel). However, taking an average of the entire tissue (as we did in Fig 7B quantifications) blunts regional anatomical differences in innervation density, which we can observe when qualitatively assessing the tissues. This can also be seen when looking at the 2D representations in Fig 7B, where the anterior and posterior portions display differences in innervation status after cold. Of note, average innervation density per inguinal scWAT depot totaled up to 25 meters per tissue depot, underscoring the great density of nerve fibers contained in WAT depots.

In separate experiments, we mimicked cold exposure by delivering the beta-3 adrenergic agonist CL316,243 (‘CL’) to wild type mice, and measured secretion of the neurotrophic factor BDNF from scWAT tissue explants. This approach revealed a significant increase in BDNF in scWAT after 10–14 days of CL treatments (Fig 7C), supporting the notion that cold stimulation increases BDNF levels in adipose, while neuropathic states reduce BDNF expression in adipose.

Since we had seen trends for decreased *Bdnf* gene expression in both obesity and age-related inguinal scWAT neuropathy (Fig 2G, S2B Fig), and an increase in BDNF protein secretion in response to noradrenergic stimulation (Fig 7C), we decided to investigate whether there were sex differences in BDNF expression in inguinal scWAT that may underlie the resistance to neuropathy in the diabetic female mice. Indeed, after a 3-day cold exposure, adult (16 weeks old) female mice (C57BL/6J) showed a much greater level of BDNF expressed in inguinal scWAT than male mice, as measured by multiplex tissue lysate ELISA (Fig 7D). At basal (room temperature conditions) gene expression of *Bdnf* is greater in inguinal scWAT of females compared to males (Fig 7E). However, following 7 days of cold exposure this effect appears blunted (Fig 7E), indicating that BDNF gene expression (mRNA) is different than BDNF protein expression and secretion. Taken together, these data fit with the apparent protection from neuropathy in female BTBR *ob/ob* mice (S1B Fig and [31]), despite females having no difference in total innervation versus males (Fig 6), providing the possibility that females have more adipose-secreted BDNF that maintains nerve integrity in neuropathic conditions.

Anatomically, the inguinal scWAT depot can be considered in 3 distinct regions, as illustrated in Fig 7F. Region 1 is the anterior side of the depot and contains major branches of the thoracoepigastric vein (TEV) (Fig 7F, blue lines/arrows); the subiliac lymph node (SiLN), often used as an orientation landmark in inguinal scWAT, is located in Region 2, as is the superficial caudal epigastric artery (SCEA, red lines/arrow); Region 3 is the posterior end of the tissue with smaller vasculature including branches of the common iliac vein. From the anterior to the posterior side of the tissue and travelling through the subiliac lymph node is a large branching network of nerves which we have called the subiliac transverse nerves (SiTN) (Fig 7F, green lines/arrows). These nerves were present in the inguinal scWAT regardless of age, exercise intervention, or cold exposure, and travelled in parallel to the main vasculature in the depot. It is important to note that these subiliac transverse nerves are not the only nerves found within the inguinal adipose depot. Similar to previous observations in BAT [42–44] and WAT [13] thin nerve fibers extend throughout the inguinal adipose tissue. These nerves have been observed running along vasculature, extending throughout the parenchymal space and wrapping around individual adipocytes, and sympathetic nerve synapsing on adipocytes has been recently proposed as the means of leptin driven lipolysis in scWAT [17].

## Discussion

Our data have demonstrated that under certain pathophysiological conditions, including aging, obesity and diabetes, WAT from humans and mice does not maintain proper innervation and undergoes a process of neurite reduction that we call ‘adipose neuropathy’. While the exact neuropathy phenotypes differed slightly between these metabolic conditions, the underlying theme was decreased adipose tissue health. In all cases, the scWAT neuropathy was accompanied by a loss of synaptic markers and a reduction in the local expression of neurotrophic factors. However, functional validation of nerve activity was not capable at this time, and thus reduction of neurite density may not represent a reduction of nerve activity in the fat. These new findings have implications for the treatment of metabolic diseases, in that re-innervating adipose tissue may be required to properly regain metabolic control. Therapeutic interventions that act to support neurite outgrowth and synapse formation on adipocytes (and potentially also vasculature and stromovascular cells) may assist with the efficacy of glucose-lowering drugs or diet and exercise interventions.

Interestingly, while diet and exercise are often prescribed in concert, despite exercise not being a very effective means to reduce appetite, the myriad health benefits of exercise may help to bolster additional weight loss strategies. This may be in part by mediating increased peripheral nerve plasticity, including in adipose depots, but this effect may be blunted with aging, as we have demonstrated here. Furthermore, we have also demonstrated that cold exposure boosts peripheral nerve plasticity in adipose, and may also be a strategy to enhance diet and exercise-based weight loss interventions, and help prevent obesity related adipose tissue neuropathy.

We have demonstrated that both mouse and human scWAT undergo neuropathy with aging and diabetes/obesity, and these data provide evidence that peripheral neuropathy is not restricted to classical tissues such as epidermis and distal limbs. Adipose tissue nerves are known to be essential for energy-expending processes such as lipolysis and thermogenesis, and loss of a proper nerve supply can have serious detrimental effects on metabolic control, which may exacerbate or initiate an insulin resistant state. Additionally, as recently demonstrated, leptin stimulated lipolysis is mediated at least in part by sympathetic activation of scWAT [17], further underscoring how adipose neuropathy can contribute to metabolic dysfunction through obesity-induced leptin resistance.

In all pathophysiological states we studied, lack of adipose innervation was associated with one or more of the following: an increase in adipose depot size, loss of proper adipose tissue function and metabolic control, or aberrant changes in gene and protein expression. Neuropathy appeared to be most severe in the obese and diabetic BTBR *ob/ob* mice, which displayed additional lack of synaptic integrity in the neuromuscular junction and BAT, as well as in skin and underlying adipose. Interestingly, gene expression of synaptic markers and neurotrophic factors was increased in iBAT of obese and diabetic BTBR *ob/ob* mice (Fig 2D and 2E), which may be an early compensatory response to decreased thermogenic function, or indicative of nerve sprouting. Bone may also be experiencing neuropathy along with a reduction in bone density, although this was not directly assessed.

In the aged mouse model, most striking was the loss of innervation around the vasculature of WAT, without any apparent deficits to the vasculature morphology or to vascular markers such as *Cd31* and *Vegfa* (at least at the mRNA level). However, these markers cannot attest to the functionality of adipose vasculature, which is not easily assessed. Interestingly, the vasculature derived neurotrophic factor NTF3 showed a trend for reduction in aged mice (S2B Fig), which may be contributing to decline of vasculature innervation with age. Aging is known to cause alterations in the structure and function of vasculature [45] and leads to decreased basal limb blood flow [46], which may result from loss of nerve supply to the blood vessels, but to our knowledge this has not yet been investigated. Exercise was not able to fully restore the innervation around the adipose vasculature in the aged mice. However, a longer period of exercise may be important for prevention of aging-related diabetes by maintaining adipose innervation and metabolic health, similarly to how exercise induces neural plasticity in the brain [47].

Beyond vasoregulation, it is still unclear exactly how nerves and blood vessels interact and whether their plasticity is functionally linked, and this warrants further investigation. Vasculature within the adipose tissue promotes metabolic health by preventing adipose tissue hypoxia and subsequent inflammation, and provides endocrine communication between adipose tissue and the rest of the body. Nerves also rely on vasculature for sustained health, since they become damaged in a hypoxic state, while vasculature relies on innervation for constriction and vasodilation of blood vessels. Since nerves, such as those in the sympathetic nervous system, regulate vascular control (ie: vasoconstriction) and may also be involved in angiogenesis, adipose neuropathy may also have adverse effects on endocrine system communication with adipose tissue and may lead to adipose hypoxia due to lack of proper vascular supply [48]. The association here could also go in the other direction, with circulating factors affecting nerve supply (ie: glucose, lipids, hormones)–but these hypotheses require closer investigation.

Synaptic markers and the neurotrophic factor BDNF were reduced with adipose neuropathy, fitting with the loss of pan-neuronal protein expression in neuropathic WAT depots. Neurotrophic factors, such as BDNF, are important mediators of neural plasticity, essential for nerve survival and growth, neurite outgrowth and branching, and synaptogenesis. Loss of neurotrophic signaling can prompt neurite retraction, axonal degeneration, and nerve death. It is well known that exercise often leads to an increase of BDNF in the brain [49, 50] and in the peripheral circulation [37], and that this improves health outcomes in patients with neurodegenerative diseases such as Parkinson’s [51]. It has been shown that BDNF is expressed in adipose tissue, but it’s role there is vastly understudied [52]. Nerve growth factor (NGF) has also been reported as a neurotrophic factor expressed in adipose tissue [53] but until recently its role in adipose tissue had been unclear. Cao *et al*. recently argued that cold induced synaptic plasticity in scWAT is dependent on adipose derived NGF [19]. Other neurotrophic factors are expressed in adipose depots but their functional significance is not currently known. Here we present a case for a potential role of BDNF in maintaining WAT peripheral nerve innervation. This idea is strengthened by the increase in adipose BDNF in response to cold exposure and exercise.

We as well as others [19, 40, 41, 54] have shown that noradrenergic stimulation increased nerve density in scWAT. Previously reported increases in sympathetic nerve fiber density in response to cold stimulation have been correlated to areas of increased browning within the adipose tissue [40, 41]. Catecholamines elicit differential effects when signaling through α- or β-adrenergic receptors (AR) in adipose. ARs belong to the family of G-protein coupled receptors (GPCRs); the five ARs active in white and brown adipose are α-1 AR (ADRA1), α-2 AR (ADRA2), and β1-, β-2, β-3, AR (ADRB1/2/3) [55]. ADRB3 is the predominate βAR present in rodent adipose, through which sympathetic nerve released neurotransmitter, norepinephrine (NE) acts [56]. βAR mediated signaling activates lipolysis through stimulating activity of adenylyl cyclase, thus increasing intracellular cyclic adenosine monophosphate (cAMP), which in turn stimulates cAMP dependent protein kinase activity that activates hormone sensitive lipase (HSL) by phosphorylation. By contrast, ADRA2 mediated signaling promotes adenylyl cyclase inhibition leading to antilipolytic effects [56].

Although catecholamines have a higher affinity for αARs than βARs, physiological effects of catecholamine signaling through ARs is largely determined by receptor abundance [55] which varies between adipose depots [57]. Catecholamine residence in peripheral tissues has been observed under certain disease states, although the mechanisms behind its occurrence have not been fully understood. In obese adipose tissue lipolytic catecholamine resistance has been associated with a decrease in βARs in adipose of human females [58], and has been attributed to decreased expression of βARs on fat cells [59, 60], as well as increased sensitivity to ADRA2 signaling [61]. There may be a similarity between lipolytic catecholamine resistance and insulin or leptin resistance, where receptors are no longer properly transducing the ligand signals.

A recently discovered subset of macrophages present in scWAT interacts with sympathetic nerves and degrades NE [62]. These sympathetic nerve associated macrophages or “SAMs” increase in abundance in obesity [62] and their increased activity of NE degradation can be seen as a type of AR ligand sequestration, possibly contributing to lipolytic catecholamine resistance in obesity. NE degrading macrophages have also been reported to increase in aging [63], and aging is associated with decreased lipolysis in adipose tissue. These reports, combined with our findings presented here, all likely contribute to reduced lipolysis under the obese state. It is unknown whether loss of innervation leads to decreased βARs in increased NE degrading macrophages in adipose tissue, or vice versa, and this requires further investigation. However, lipolysis can also be affected by non-adrenergic signaling as recently reviewed [64] and should be considered. Purinergic (via adenosine and ATP) signaling in adipose produces similarly diverse effects as NE signaling and is dependent on specific receptor activation. Adenosine appears to be a negative modulator of lipolysis in WAT, yet has pro-lipolytic activity in BAT [64]. Other neurotransmitters and neuropeptides are expressed in adipose tissues, such as, NPY, Substance P, CGRP and others [48]. It is currently unknown what role, if any, these play in lipolysis or other adipose tissue functions.

Finally, we have revealed that iBAT and scWAT differ in their patterns of innervation and response to neuropathic stimuli, and that male and female mice differ in scWAT innervation with a blunted response to neuropathy and higher levels of BDNF. Using a new technique for whole-depot imaging of adipose innervation, we have also revealed a consistent pattern of nerves in inguinal WAT (traversing the subiliac lymph node), which changes regionally in response to cold stimulation.

Taken together, we have demonstrated for the first time that adipose tissue nerves are able to undergo neuropathy in pathophysiological situations, but can also undergo neural plasticity in response to exercise or cold exposure. We have also identified BDNF as a locally produced peripheral nerve survival factor in WAT. Changes in adipose innervation status were correlated with altered metabolic control and underscore the importance of adipose nerves for proper metabolic health.

## Supporting information

S1 Table Human cohort data by age. (DOCX)

S2 Table Human characteristic data. (DOCX)

S3 Table Human cohort data total statistics. (DOCX)

S4 Table Raw human data. (XLSX)

S5 Table qPCR primers. (DOCX)

S6 Table General antibody table. (XLSX)

S1 File Uncropped western blots. (PDF)

S1 Fig Adiposity and neuropathy of BTBR *ob/ob* mutant (MUT) mice. Male and female BTBR MUT mice were assessed for total body weight and adiposity (inguinal scWAT/body weight), and compared to WT or HET animals in a pilot cohort (a). Female BTBR WT, HET, and MUT were assessed for tactile allodynia via the Von Frey assay, an indirect measure of peripheral neuropathy (b). Protein expression of PGP9.5 (c) and TH (d) in inguinal scWAT of HET, MUT, and WT BTBR mice was measured via western blotting. Protein expression was normalized to Cyclophilin B and band density was quantified in Image J and analyzed using a two-tailed Student’s t-test. Error bars are SEMs. For (a-d), males: MUT N = 2; WT N = 3; HET N = 3; females: MUT: N = 1; WT: N = 3; HET: N = 3; all mice were 12–24 weeks old. Data represents a pilot cohort to compare HET to MUT mice, and males to females, thus statistical analyses were not performed due to small sample sizes. Sample Ponceau S staining on blot corresponding to Fig 1F demonstrates equal protein loading (e).
(TIF)

S2 Fig Innervation of vasculature in young sedentary (sed) and exercised (run) mice. Gene expression analysis of axillary scWAT from young (10–12 week old) sedentary (young sed) versus aged (16 month old) sedentary (aged sed) male mice for vascular markers (a) and neurotrophic factors (b). Gene expression was analyzed by two-tailed Student’s t-test, N = 4 for sedentary and N = 5 for run. Error bars are SEMs. vascular markers. Whole depot nerve and vasculature imaging of inguinal scWAT from exercise (run) animals was performed by combining immunostaining for PGP9.5 (green) with autofluorescence of vasculature (red/orange); images were captured at 10x on Nikon Eclipse E400 microscope (c). Images are representative of N = 5 mice analyzed per group. Body weight, adiposity (inguinal scWAT/body weight & pgWAT/body weight), and quadricep muscle weight was measured for young (12–15 week old) mice under sedentary (sed) and exercised (run) conditions (d). Body and tissue weight analyzed by one-way ANOVA with Tukey post hoc. N = 4 for sedentary and N = 5 for run groups. Protein expression for PSD95 in axillary scWAT was determined by western blotting (e). Protein expression was normalized to β-Tubulin; band density was quantified in Image J and analyzed by two-tailed Student’s t-test. Gene expression analysis of inguinal scWAT from young (12–15 week old) sedentary (young sed) versus young exercised (young run) male mice (f), gene expression was analyzed by two-tailed Student’s t-test, N = 4 for sedentary and N = 5 for run. Error bars are SEMs. Whole inguinal scWAT depots were collected from young *C57BL6/J* mice under sedentary and exercised (run) conditions. Tissue was stained with PGP9.5 (green) and Isolectin IB-4 (red) to analyze nerve and blood vessel interactions in WAT with exercise. Tissues were scanned at 10x for blood vessels 50um or greater in diameter. Up to twenty blood vessels were evaluated for innervation in each tissue (g). Percentage of innervated vessels with a diameter of ≥ 50um were evaluated per tissue (g, left panel). Analyzed with two-tailed Student’s T-Test. Error bars are SEMs. Innervation was further characterized by the number of resident nerves per blood vessel (g, right panel). Data was analyzed using a two-way ANOVA and Tukey’s multiple comparison test. Error bars are SEMs. Fluorescent imaging of blood vessel and nerve interactions at 4x, 10x, and 40x using a Nikon Eclipse E400 microscope (h).
(TIF)

## References

[1] Ryu V, Watts AG, Xue B, Bartness TJ. Bidirectional crosstalk between the sensory and sympathetic motor systems innervating brown and white adipose tissue in male Siberian hamsters. American journal of physiology Regulatory, integrative and comparative physiology. 2017;312(3):R324–r37. Epub 2017/01/13. 10.1152/ajpregu.00456.2015 28077392 PMC5401994

[2] Morrison SF, Madden CJ. Central nervous system regulation of brown adipose tissue. Comprehensive Physiology. 2014;4(4):1677–713. Epub 2014/11/28. 10.1002/cphy.c140013 25428857 PMC4435534

[3] Bartness TJ, Shrestha YB, Vaughan CH, Schwartz GJ, Song CK. Sensory and sympathetic nervous system control of white adipose tissue lipolysis. Mol Cell Endocrinol. 2010;318(1–2):34–43. Epub 2009/09/15. 10.1016/j.mce.2009.08.031 19747957 PMC2826518

[4] Bartness TJ, Vaughan CH, Song CK. Sympathetic and sensory innervation of brown adipose tissue. Int J Obes (Lond). 2010;34 Suppl 1:S36–42.:S36-S42. 20935665 10.1038/ijo.2010.182 PMC3999344

[5] Townsend KL, Tseng YH. Brown fat fuel utilization and thermogenesis. Trends Endocrinol Metab. 2014;25(4):168–77. Epub 2014/01/07. 10.1016/j.tem.2013.12.004 24389130 PMC3972344

[6] Ramseyer VD, Granneman JG. Adrenergic regulation of cellular plasticity in brown, beige/brite and white adipose tissues. Adipocyte. 2016;5(2):119–29. Epub 2016/07/08. 10.1080/21623945.2016.1145846 27386156 PMC4916885

[7] Geloen A, Collet AJ, Bukowiecki LJ. Role of sympathetic innervation in brown adipocyte proliferation. Am J Physiol. 1992;263(6 Pt 2):R1176–R81. 1481924 10.1152/ajpregu.1992.263.6.R1176

[8] Desautels M, Dulos RA, Mozaffari B. Selective loss of uncoupling protein from mitochondria of surgically denervated brown adipose tissue of cold-acclimated mice. BiochemCell Biol. 1986;64(11):1125–34. 10.1139/o86-148 3828106

[9] Bowers RR, Festuccia WT, Song CK, Shi H, Migliorini RH, Bartness TJ. Sympathetic innervation of white adipose tissue and its regulation of fat cell number. American journal of physiology Regulatory, integrative and comparative physiology. 2004;286(6):R1167–75. Epub 2004/05/15. 10.1152/ajpregu.00558.2003 . 15142857

[10] Cantu RC, Goodman HM. Effects of denervation and fasting on white adipose tissue. Am J Physiol. 1967;212(1):207–12. Epub 1967/01/01. . 6016006 10.1152/ajplegacy.1967.212.1.207

[11] Shi H, Song CK, Giordano A, Cinti S, Bartness TJ. Sensory or sympathetic white adipose tissue denervation differentially affects depot growth and cellularity. American journal of physiology Regulatory, integrative and comparative physiology. 2005;288(4):R1028–37. Epub 2004/11/20. 10.1152/ajpregu.00648.2004 . 15550613

[12] Bartness TJ, Song CK. Brain-adipose tissue neural crosstalk. Physiol Behav. 2007;91(4):343–51. 10.1016/j.physbeh.2007.04.002 17521684 PMC1986714

[13] Giordano A, Morroni M, Santone G, Marchesi GF, Cinti S. Tyrosine hydroxylase, neuropeptide Y, substance P, calcitonin gene-related peptide and vasoactive intestinal peptide in nerves of rat periovarian adipose tissue: an immunohistochemical and ultrastructural investigation. J Neurocytol. 1996;25(2):125–36. Epub 1996/02/01. . 8699194 10.1007/BF02284791

[14] Blaszkiewicz M, Townsend KL. Adipose Tissue and Energy Expenditure: Central and Peripheral Neural Activation Pathways. Current obesity reports. 2016;5(2):241–50. Epub 2016/04/09. 10.1007/s13679-016-0216-9 . 27055864

[15] Giordano A, Frontini A, Castellucci M, Cinti S. Presence and distribution of cholinergic nerves in rat mediastinal brown adipose tissue. J Histochem Cytochem. 2004;52(7):923–30. Epub 2004/06/23. 10.1369/jhc.3A6246.2004 . 15208359

[16] Bartness TJ, Vaughan CH, Song CK. Sympathetic and sensory innervation of brown adipose tissue. Int J Obes (Lond). 2010;34 Suppl 1:S36–42. Epub 2010/10/12. 10.1038/ijo.2010.182 20935665 PMC3999344

[17] Zeng W, Pirzgalska RM, Pereira MM, Kubasova N, Barateiro A, Seixas E, et al Sympathetic neuro-adipose connections mediate leptin-driven lipolysis. Cell. 2015;163(1):84–94. Epub 2015/09/26. 10.1016/j.cell.2015.08.055 . 26406372 PMC7617198

[18] Jiang H, Ding X, Cao Y, Wang H, Zeng W. Dense Intra-adipose Sympathetic Arborizations Are Essential for Cold-Induced Beiging of Mouse White Adipose Tissue. Cell Metab. 2017;26(4):686–92 e3. Epub 2017/09/19. 10.1016/j.cmet.2017.08.016 . 28918935

[19] Cao Y, Wang H, Zeng W. Whole-tissue 3D imaging reveals intra-adipose sympathetic plasticity regulated by NGF-TrkA signal in cold-induced beiging. Protein Cell. 2018;9(6):527–39. Epub 2018/03/29. 10.1007/s13238-018-0528-5 29589323 PMC5966360

[20] Hanewinckel R, Drenthen J, van Oijen M, Hofman A, van Doorn PA, Ikram MA. Prevalence of polyneuropathy in the general middle-aged and elderly population. Neurology. 2016;87(18):1892–8. Epub 2016/11/02. 10.1212/wnl.0000000000003293 . 27683845

[21] Tesfaye S, Stevens LK, Stephenson JM, Fuller JH, Plater M, Ionescu-Tirgoviste C, et al Prevalence of diabetic peripheral neuropathy and its relation to glycaemic control and potential risk factors: the EURODIAB IDDM Complications Study. Diabetologia. 1996;39(11):1377–84. Epub 1996/11/01. . 8933008 10.1007/s001250050586

[22] Kaley TJ, Deangelis LM. Therapy of chemotherapy-induced peripheral neuropathy. Br J Haematol. 2009;145(1):3–14. Epub 2009/01/28. 10.1111/j.1365-2141.2008.07558.x . 19170681

[23] Vileikyte L, Rubin RR, Leventhal H. Psychological aspects of diabetic neuropathic foot complications: an overview. Diabetes Metab Res Rev. 2004;20 Suppl 1:S13–8. Epub 2004/05/20. 10.1002/dmrr.437 . 15150807

[24] Vinik AI, Park TS, Stansberry KB, Pittenger GL. Diabetic neuropathies. Diabetologia. 2000;43(8):957–73. 10.1007/s001250051477 10990072

[25] DeFronzo RA. Glucose intolerance and aging. Diabetes Care. 1981;4(4):493–501. 7049632 10.2337/diacare.4.4.493

[26] Barzilai N, Banerjee S, Hawkins M, Chang CJ, Chen W, Rossetti L. The effect of age-dependent increase in fat mass on peripheral insulin action is saturable. J GerontolABiol Sci Med Sci. 1998;53(2):B141–B6. 10.1093/gerona/53a.2.b141 9520910

[27] Cartwright MJ, Tchkonia T, Kirkland JL. Aging in adipocytes: potential impact of inherent, depot-specific mechanisms. Exp Gerontol. 2007;42(6):463–71. 10.1016/j.exger.2007.03.003 17507194 PMC1961638

[28] Anish L, Nagappa M, Mahadevan A, Taly AB. Neuropathy in elderly: lessons learnt from nerve biopsy. Age and ageing. 2015;44(2):312–7. Epub 2014/11/05. 10.1093/ageing/afu171 . 25362502

[29] O'Brien PD, Hur J, Hayes JM, Backus C, Sakowski SA, Feldman EL. BTBR ob/ob mice as a novel diabetic neuropathy model: Neurological characterization and gene expression analyses. Neurobiology of disease. 2015;73:348–55. Epub 2014/12/03. 10.1016/j.nbd.2014.10.015 25447227 PMC4416075

[30] Cheng HT, Dauch JR, Hayes JM, Hong Y, Feldman EL. Nerve growth factor mediates mechanical allodynia in a mouse model of type 2 diabetes. J Neuropathol Exp Neurol. 2009;68(11):1229–43. Epub 2009/10/10. 10.1097/NEN.0b013e3181bef710 19816194 PMC3163104

[31] O’Brien PD, Hur J, Robell NJ, Hayes JM, Sakowski SA, Feldman EL. Gender-specific differences in diabetic neuropathy in BTBR ob/ob mice. J Diabetes Complications. 2016;30(1):30–7. 10.1016/j.jdiacomp.2015.09.018 26525588 PMC4698064

[32] Klingenspor M, Meywirth A, Stohr S, Heldmaier G. Effect of unilateral surgical denervation of brown adipose tissue on uncoupling protein mRNA level and cytochrom-c-oxidase activity in the Djungarian hamster. Journal of comparative physiology B, Biochemical, systemic, and environmental physiology. 1994;163(8):664–70. Epub 1994/01/01. . 8195470 10.1007/BF00369517

[33] Hamilton JM, Bartness TJ, Wade GN. Effects of norepinephrine and denervation on brown adipose tissue in Syrian hamsters. Am J Physiol. 1989;257(2 Pt 2):R396–404. Epub 1989/08/01. . 2548410 10.1152/ajpregu.1989.257.2.R396

[34] Rix M, Andreassen H, Eskildsen P. Impact of peripheral neuropathy on bone density in patients with type 1 diabetes. Diabetes Care. 1999;22(5):827–31. Epub 1999/05/20. 10.2337/diacare.22.5.827 . 10332690

[35] Kim JH, Jung MH, Lee JM, Son HS, Cha BY, Chang SA. Diabetic peripheral neuropathy is highly associated with nontraumatic fractures in Korean patients with type 2 diabetes mellitus. Clin Endocrinol (Oxf). 2012;77(1):51–5. Epub 2011/09/13. 10.1111/j.1365-2265.2011.04222.x . 21906118

[36] Kingsley K, Carroll K, Huff JL, Plopper GE. Photobleaching of arterial autofluorescence for immunofluorescence applications. Biotechniques. 2001;30(4):794–7. Epub 2001/04/21. . 11314262 10.2144/01304st05

[37] Fiuza-Luces C, Garatachea N, Berger NA, Lucia A. Exercise is the real polypill. Physiology (Bethesda, Md). 2013;28(5):330–58. Epub 2013/09/03. 10.1152/physiol.00019.2013 . 23997192

[38] Gamu D, Trinh A, Bombardier E, Tupling AR. Persistence of diet-induced obesity despite access to voluntary activity in mice lacking sarcolipin. Physiol Rep. 2015;3(9). Epub 2015/09/25. 10.14814/phy2.12549 26400985 PMC4600390

[39] Gurley JM, Griesel BA, Olson AL. Increased Skeletal Muscle GLUT4 Expression in Obese Mice After Voluntary Wheel Running Exercise Is Posttranscriptional. Diabetes. 2016;65(10):2911–9. Epub 2016/07/15. 10.2337/db16-0305 27411383 PMC5033261

[40] Vitali A, Murano I, Zingaretti MC, Frontini A, Ricquier D, Cinti S. The adipose organ of obesity-prone C57BL/6J mice is composed of mixed white and brown adipocytes. J Lipid Res. 2012;53(4):619–29. Epub 2012/01/25. 10.1194/jlr.M018846 22271685 PMC3307639

[41] Murano I, Barbatelli G, Giordano A, Cinti S. Noradrenergic parenchymal nerve fiber branching after cold acclimatisation correlates with brown adipocyte density in mouse adipose organ. Journal of anatomy. 2009;214(1):171–8. 10.1111/j.1469-7580.2008.01001.x 19018882 PMC2667925

[42] Sidman RL, Fawcett DW. The effect of peripheral nerve section on some metabolic responses of brown adipose tissue in mice. The Anatomical Record. 1954;118(3):487–507. 10.1002/ar.1091180303. 13158866

[43] Wirsen C. Distribution of adrenergic nerve fibers in brown and white adipose tissue Handbook of physiology: American Physiological Society, Washington, DC; 1965 p. 197–9.

[44] De Matteis R, Ricquier D, Cinti S. TH-, NPY-, SP-, and CGRP-immunoreactive nerves in interscapular brown adipose tissue of adult rats acclimated at different temperatures: an immunohistochemical study. J Neurocytol. 1998;27(12):877–86. Epub 2000/02/05. . 10659680 10.1023/a:1006996922657

[45] Scioli MG, Bielli A, Arcuri G, Ferlosio A, Orlandi A. Ageing and microvasculature. Vasc Cell. 2014;6:19 Epub 2014/09/23. 10.1186/2045-824X-6-19 25243060 PMC4169693

[46] Dinenno FA, Tanaka H, Stauffer BL, Seals DR. Reductions in basal limb blood flow and vascular conductance with human ageing: role for augmented alpha-adrenergic vasoconstriction. J Physiol. 2001;536(Pt 3):977–83. Epub 2001/11/03. 10.1111/j.1469-7793.2001.00977.x 11691889 PMC2278891

[47] Wolf SA, Melnik A, Kempermann G. Physical exercise increases adult neurogenesis and telomerase activity, and improves behavioral deficits in a mouse model of schizophrenia. Brain BehavImmun. 2011;25(5):971–80. 10.1016/j.bbi.2010.10.014 20970493

[48] Blaszkiewicz M, Willows JW, Johnson CP, Townsend KL. The Importance of Peripheral Nerves in Adipose Tissue for the Regulation of Energy Balance. Biology (Basel). 2019;8(1). Epub 2019/02/15. 10.3390/biology8010010 . 30759876 PMC6466238

[49] Isackson PJ, Huntsman MM, Murray KD, Gall CM. BDNF mRNA expression is increased in adult rat forebrain after limbic seizures: temporal patterns of induction distinct from NGF. Neuron. 1991;6(6):937–48. Epub 1991/06/01. . 2054188 10.1016/0896-6273(91)90234-q

[50] Neeper SA, Gomez-Pinilla F, Choi J, Cotman C. Exercise and brain neurotrophins. Nature. 1995;373(6510):109 Epub 1995/01/12. 10.1038/373109a0 . 7816089

[51] Zoladz JA, Majerczak J, Zeligowska E, Mencel J, Jaskolski A, Jaskolska A, et al Moderate-intensity interval training increases serum brain-derived neurotrophic factor level and decreases inflammation in Parkinson's disease patients. Journal of physiology and pharmacology: an official journal of the Polish Physiological Society. 2014;65(3):441–8. Epub 2014/06/17. . 24930517

[52] Hausman GJ, Poulos SP, Richardson RL, Barb CR, Andacht T, Kirk HC, et al Secreted proteins and genes in fetal and neonatal pig adipose tissue and stromal-vascular cells. J Anim Sci. 2006;84(7):1666–81. Epub 2006/06/16. 10.2527/jas.2005-539 . 16775050

[53] Ryan VH, German AJ, Wood IS, Hunter L, Morris P, Trayhurn P. NGF gene expression and secretion by canine adipocytes in primary culture: upregulation by the inflammatory mediators LPS and TNFalpha. Horm Metab Res. 2008;40(12):861–8. Epub 2008/09/17. 10.1055/s-0028-1083782 . 18792883

[54] Townsend KL, Madden CJ, Blaszkiewicz M, McDougall L, Tupone D, Lynes MD, et al Reestablishment of Energy Balance in a Male Mouse Model With POMC Neuron Deletion of BMPR1A. Endocrinology. 2017;158(12):4233–45. 10.1210/en.2017-00212 29040444 PMC5711385

[55] Boucher J, Castan-Laurell I, Le Lay S, Grujic D, Sibrac D, Krief S, et al Human alpha 2A-adrenergic receptor gene expressed in transgenic mouse adipose tissue under the control of its regulatory elements. J Mol Endocrinol. 2002;29(2):251–64. 12370125 10.1677/jme.0.0290251

[56] Lafontan M, Berlan M. Fat cell adrenergic receptors and the control of white and brown fat cell function. J Lipid Res. 1993;34(7):1057–91. Epub 1993/07/01. . 8371057

[57] Jensen MD. Lipolysis: contribution from regional fat. Annu Rev Nutr. 1997;17:127–39. Epub 1997/01/01. 10.1146/annurev.nutr.17.1.127 . 9240922

[58] Reynisdottir S, Wahrenberg H, Carlstrom K, Rossner S, Arner P. Catecholamine resistance in fat cells of women with upper-body obesity due to decreased expression of beta 2-adrenoceptors. Diabetologia. 1994;37(4):428–35. Epub 1994/04/01. . 8063046 10.1007/BF00408482

[59] Lonnqvist F, Wahrenberg H, Hellstrom L, Reynisdottir S, Arner P. Lipolytic catecholamine resistance due to decreased beta 2-adrenoceptor expression in fat cells. J Clin Invest. 1992;90(6):2175–86. Epub 1992/12/01. 10.1172/JCI116103 1334970 PMC443368

[60] Faulds G, Ryden M, Ek I, Wahrenberg H, Arner P. Mechanisms behind lipolytic catecholamine resistance of subcutaneous fat cells in the polycystic ovarian syndrome. J Clin Endocrinol Metab. 2003;88(5):2269–73. Epub 2003/05/03. 10.1210/jc.2002-021573 . 12727985

[61] Hellström L, Rössner S, Hagström-Toft E, Reynisdottir S. Lipolytic catecholamine resistance linked to α2-adrenoceptor sensitivity—a metabolic predictor of weight loss in obese subjects. International Journal Of Obesity. 1997;21:314 10.1038/sj.ijo.0800407 9130030

[62] Pirzgalska RM, Seixas E, Seidman JS, Link VM, Sánchez NM, Mahú I, et al Sympathetic neuron–associated macrophages contribute to obesity by importing and metabolizing norepinephrine. Nature medicine. 2017;23(11):1309 10.1038/nm.4422 29035364 PMC7104364

[63] Camell CD, Sander J, Spadaro O, Lee A, Nguyen KY, Wing A, et al Inflammasome-driven catecholamine catabolism in macrophages blunts lipolysis during ageing. Nature. 2017;550(7674):119–23. Epub 2017/09/28. 10.1038/nature24022 28953873 PMC5718149

[64] Braun K, Oeckl J, Westermeier J, Li Y, Klingenspor M. Non-adrenergic control of lipolysis and thermogenesis in adipose tissues. J Exp Biol. 2018;221(Pt Suppl 1). Epub 2018/03/09. 10.1242/jeb.165381 . 29514884

[65] Kodama T, Matsuki D, Tada A, Takeda K, Mori S. New concept for the prevention and treatment of metastatic lymph nodes using chemotherapy administered via the lymphatic network. Sci Rep. 2016;6:32506 Epub 2016/09/02. 10.1038/srep32506 27581921 PMC5007471

[66] Kochi T, Imai Y, Takeda A, Watanabe Y, Mori S, Tachi M, et al Characterization of the arterial anatomy of the murine hindlimb: functional role in the design and understanding of ischemia models. PLoS One. 2013;8(12):e84047 Epub 2014/01/05. 10.1371/journal.pone.0084047 24386328 PMC3875518

