# Supplementary material for: Neuropathy and neural plasticity in the subcutaneous white adipose depot
Source: PLoS One. 2019 Sep 11;14(9):e0221766. doi: 10.1371/journal.pone.0221766 (PMC6738614; doi:10.1371/journal.pone.0221766)
Supplement: S2 Table — (DOCX) [file pone.0221766.s002.docx]

**S2 Table. Human Characteristic Data^[[1]](#footnote-1)^**

| **Used in Figure #** | **Sample #** | **Tissue** | **Sex** | **Procedure** | **Race/ Ethnicity** | **Diabetes** |
| --- | --- | --- | --- | --- | --- | --- |
| 3e-g | 1053 | sqWAT | F | Panniculectomy | Hispanic | Non-diabetic |
| 3a-c, 3e-f | 1063 | sqWAT | F | Panniculectomy | Hispanic | Non-diabetic |
| 3e-g | 1087 | sqWAT | F | Panniculectomy | African American | Diabetic |
| 3e-g | 1110 | sqWAT | F | Panniculectomy | Hispanic | Non-diabetic |
| 3a-c | 1245 | sqWAT | F | Panniculectomy | Hispanic | Non-diabetic |
| 3a-c | 1252 | sqWAT | F | Panniculectomy | African American | Non-diabetic |
| 3e-g | 1259 | sqWAT | F | Panniculectomy | Unknown | Pre-diabetic |
| 3a-c | 1262 | sqWAT | F | Panniculectomy | Hispanic | Non-diabetic |
| 3a-c | 1266 | sqWAT | F | Abdominoplasty | Unknown | Non-diabetic |
| 3e-g | 1271 | sqWAT | F | Abdominoplasty | Hispanic | Non-diabetic |
| 3e | 1046 | sqWAT | F | Panniculectomy | African American | Diabetic |
| 3a-c | 1054 | sqWAT | F | Panniculectomy | Hispanic | Diabetic |
| 3a-c | 1104 | sqWAT | F | Panniculectomy | African American | Diabetic |
| 3a-d, 3h | 1061 | sqWat, Omental | F | Gastric Bypass | African American | Non-diabetic |
| 3a-h | 1066 | sqWat, Omental | F | Gastric Bypass | Unknown | Non-diabetic |
| 3b-h | 1083 | sqWat, Omental | F | Gastric Bypass | Hispanic | Non-diabetic |
| 3d, 3h | 1273 | Omental | F | Gastric Bypass | Hispanic | Non-diabetic |
| 3d, 3h | 1251 | Omental | M | Gastrectomy Sleeve | Hispanic | Non-diabetic |
| 3d, 3h | 1272 | Omental | F | Gastric Bypass | Hispanic | Pre-diabetic |
| 3d, 3h | 1247 | Omental | F | Gastric Bypass | Hispanic | Non-diabetic |

1. Patient tissue type analyzed, sex, elective surgical procedure, race or ethnicity, and diabetic state are presented along with which figure samples were used in. sqWAT = subcutaneous white adipose tissue; BMI = body mass index; F = female; M = male. [↑](#footnote-ref-1)
