# Supplementary material for: Neuropathy and neural plasticity in the subcutaneous white adipose depot
Source: PLoS One. 2019 Sep 11;14(9):e0221766. doi: 10.1371/journal.pone.0221766 (PMC6738614; doi:10.1371/journal.pone.0221766)
Supplement: S5 Table — (DOCX) [file pone.0221766.s005.docx]

**S5 Table. qPCR Primers**

| **qPCR Primers** | | |
| --- | --- | --- |
| ***Gene*** | **Forward Sequence** | **Reverse Sequence** |
| *cd31* | ACGCTGGTGCTCTATGCAAG | TCAGTTGCTGCCCATTCATCA |
| *cidea* | ATCACAACTGGCCTGGTTACG | TACTACCCGGTGTCCATTTCT |
| *dio2* | CAGTGTGGTGCACTGCTCCAATC | TGAACCAAAGTTGACCACCAG |
| *il1β* | GAAATGCCACCTTTTGACAGTG | TGGATGCTCTCATCAGGACAG |
| *il4* | GGTCAACCCCCAGCTAGT | GCCGATGATCTCTCTCAAGTGAT |
| *Il6* | TAGTCCTTCCTACCCCAATTTC | TTGGTCCTTAGCCACTCCTTC |
| *il10* | CTATGCTGCCTGCTCTTACTGAC | CGGAGAGAGGTACAAACGAGG |
| *il13* | CCTGGCTCTTGCTTGCCTT | GGTCTTGTGTGATGTTGCTCA |
| *pgc1α* | CCCTGCCATTGTTAAGACC | TGCTGCTGTTCCTGTTTTC |
| *psd95* | GCGGTGCTAAAATCGAATGC | ACAGAGAGGGGCAGGCAGT |
| *sox10* | AGATCCAGTTCCGTGTCAATAA | GCGAGAAGAAGGCTAGGTG |
| *synapsin I* | CATGGCACGTAATGGAGACTACCGCA | CCGCCAGCATGCCTTC |
| *synapsin II* | GCCACCAGGTTAAGCTCTGA | TTCCAGGAAGGCCAAGGT |
| *synaptophysin* | TGACTTCAGGACTCAACACCTC | CAGGAGCTGGTTGCTTTTCT |
| *tnfα* | GGGCCACCACGCTCTTCTGTCT | GCCACTCCAGCTGCTCCTCCAC |
| *ucp1* | AGGCTTCCAGTACCATTAGGT | CTGAGTGAGGCAAAGCTGATTT |
| *vegfa* | AACAAAGCCAGAAAATCACTGTGA | CGGATCTTGGACAAACAAATGC |
