# Supplementary material for: Neuropathy and neural plasticity in the subcutaneous white adipose depot
Source: PLoS One. 2019 Sep 11;14(9):e0221766. doi: 10.1371/journal.pone.0221766 (PMC6738614; doi:10.1371/journal.pone.0221766)

Note

- All uncropped/unedited blots associated with our western blot data for this manuscript are included here
- Please note that if certain samples are precious and needed for multiple antibodies, we sometimes cut the blot BEFORE we do primary antibody incubations – so, some blots are full size and some are half size in these images
- These images were captured on a Syngene G:Box using standardized imaging protocols developed for our laboratory.

Blot associated with Fig 1d.

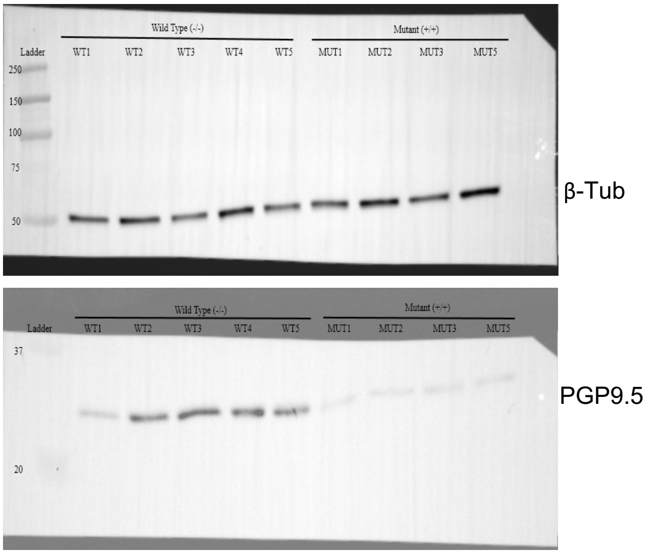

Blot associated with Fig 1e.

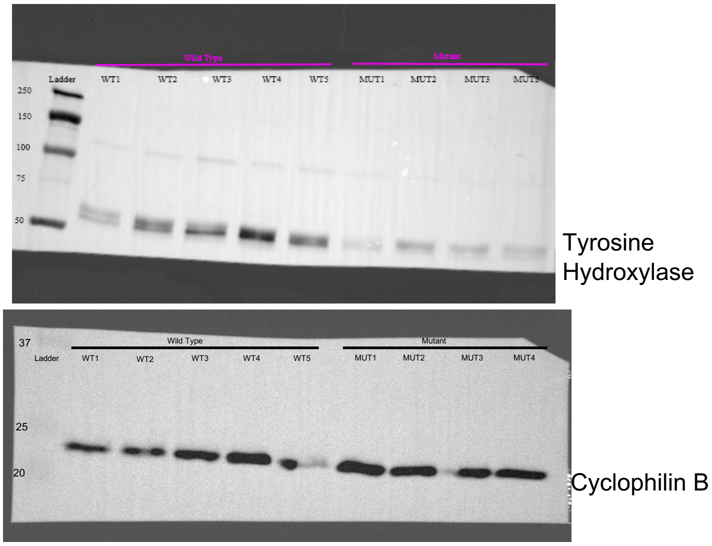

Blot associated with Fig 1f.

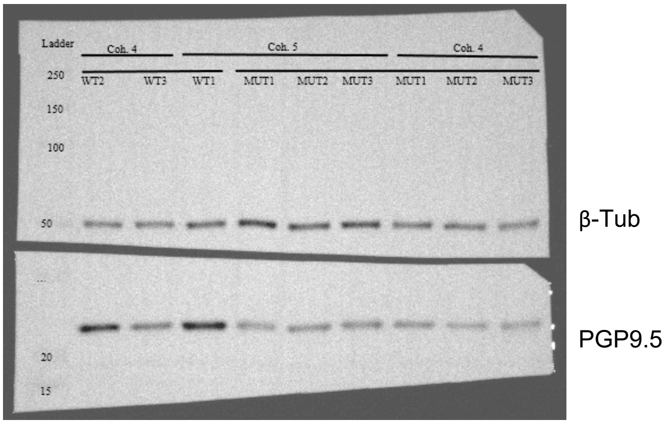

Blot associated with Fig 1g.

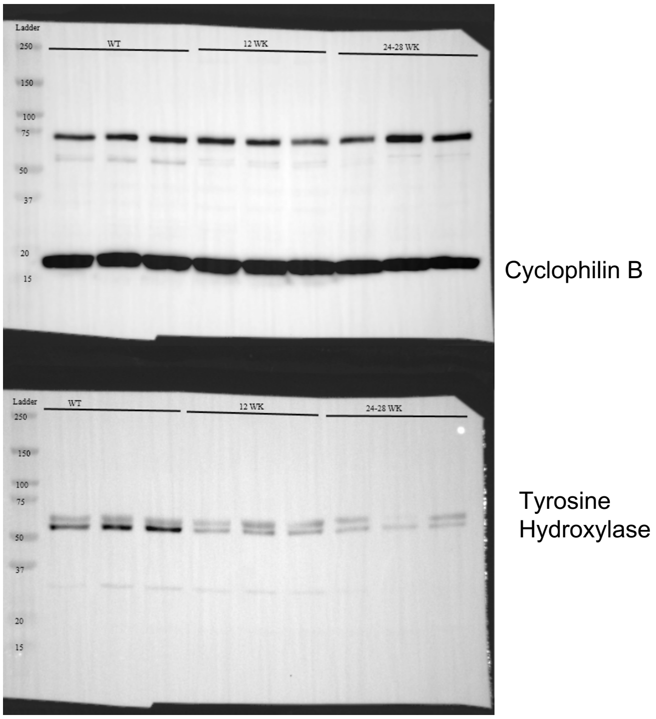

Blot associated with Fig 1h.

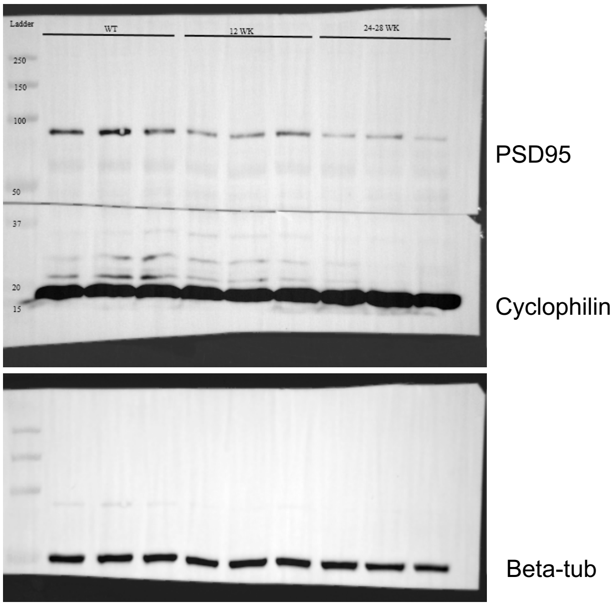

Blot associated with Fig 1i.

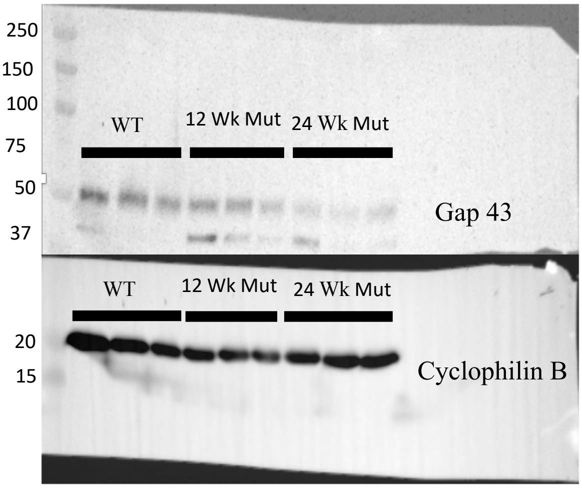

Blot associated with Fig 2a.

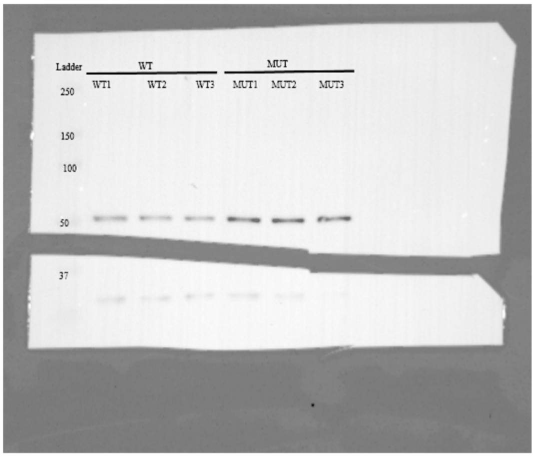

β-tubulin

PGP9.5

Blot associated with Fig 2b.

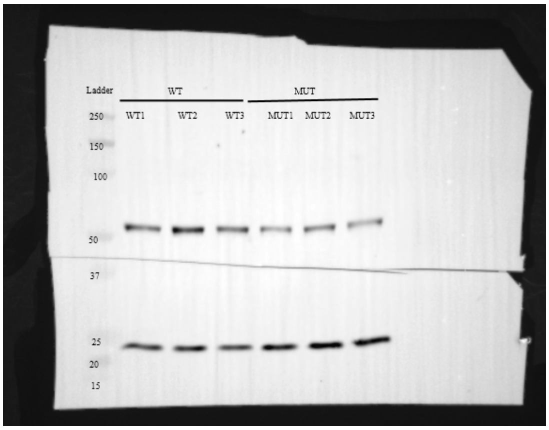

Tyrosine Hydroxylase

Cyclophilin B

Blot associated with Fig 3a.

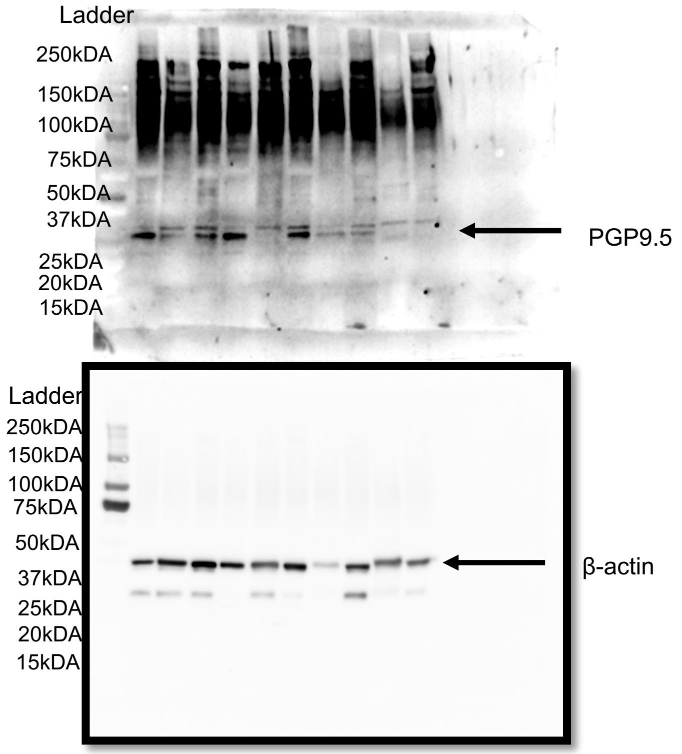

Blot associated with fig 3d.

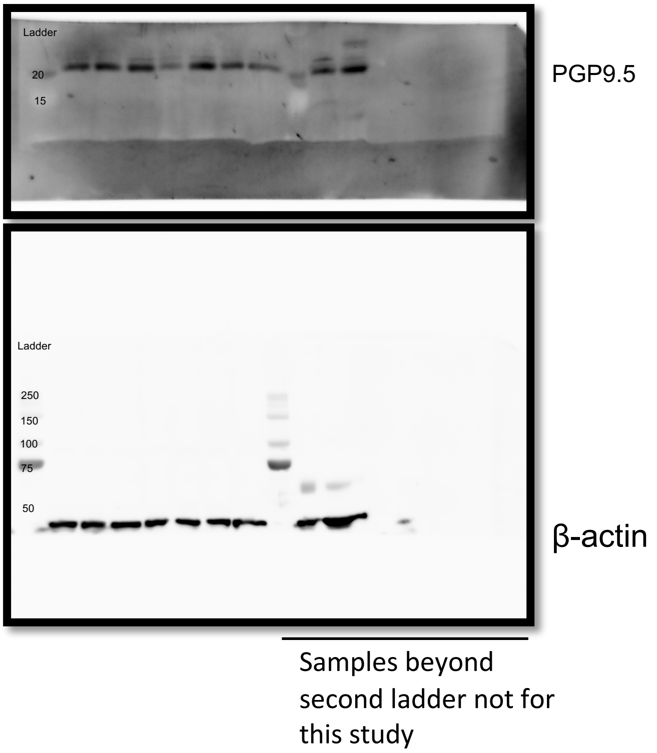

Blot associated with Fig 3e.

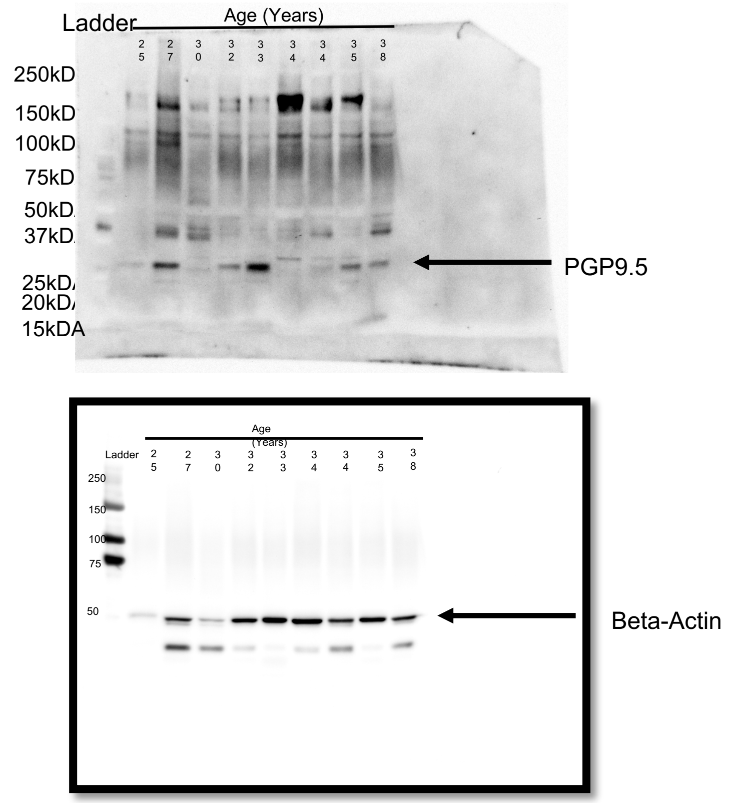

Blot associated with Fig 3f.

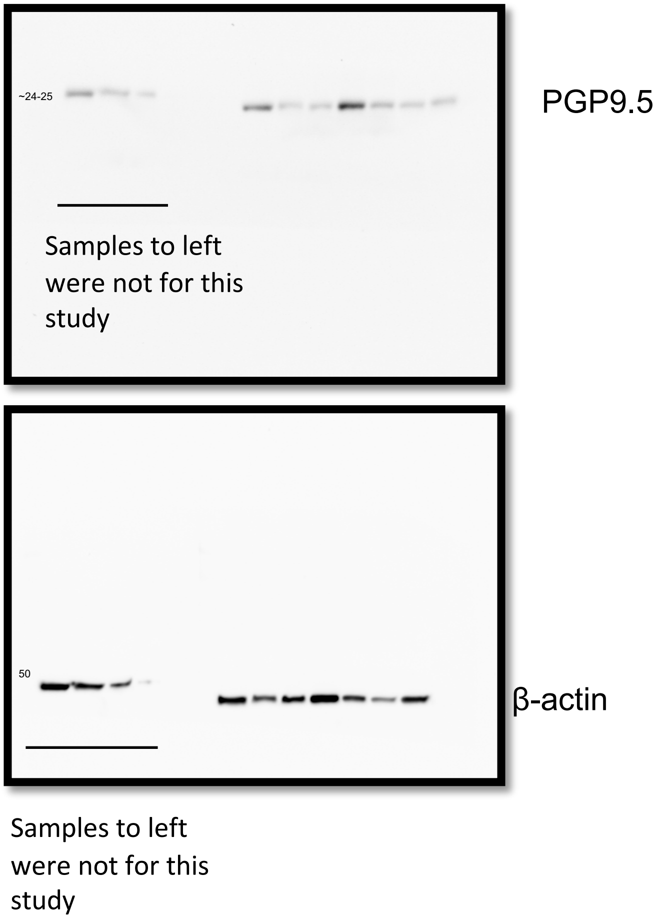

Blot Associated with Fig 4a.

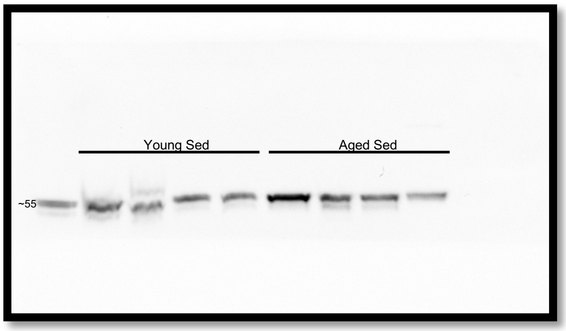

Beta-tubulin

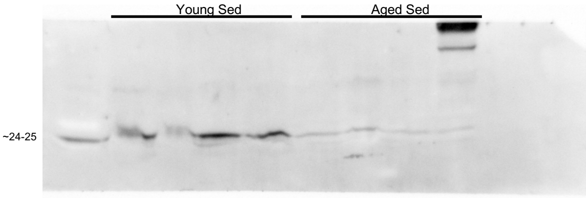

PGP9.5

Blot associated with Fig 4b.

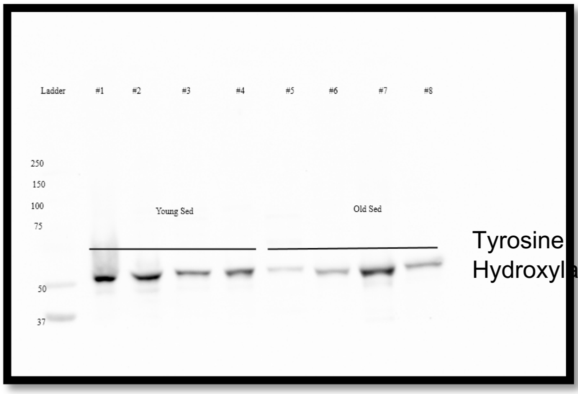

Tyrosine Hydroxylase

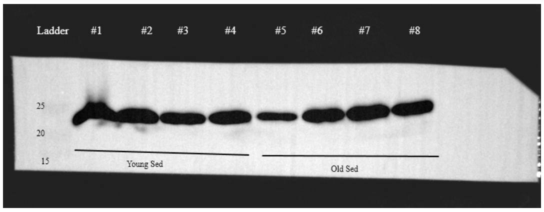

Cyclophilin B

Blot Associated with Fig 4c.

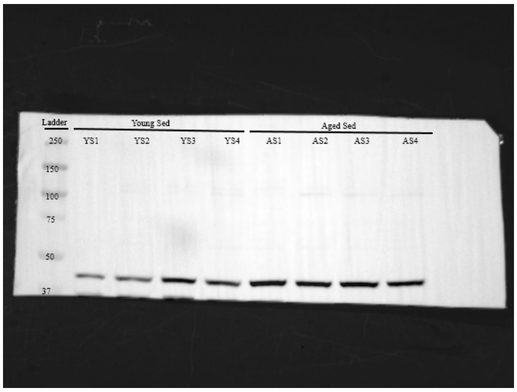

Beta- actin

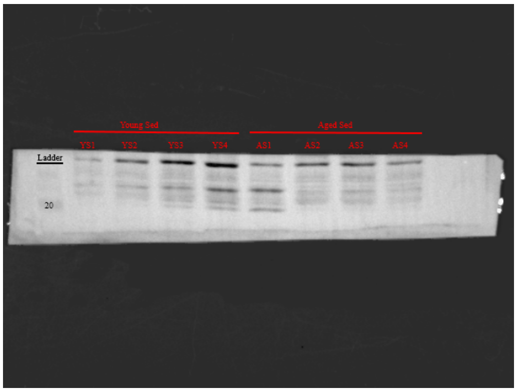

PGP9.5

Blot blot associated with fig 4d.

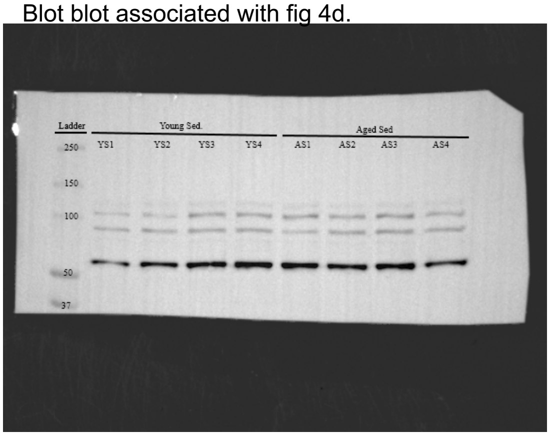

TH

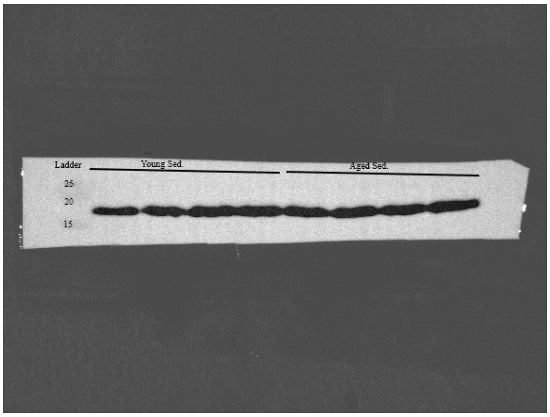

Cyclo b

Blot associated with Fig 5a.

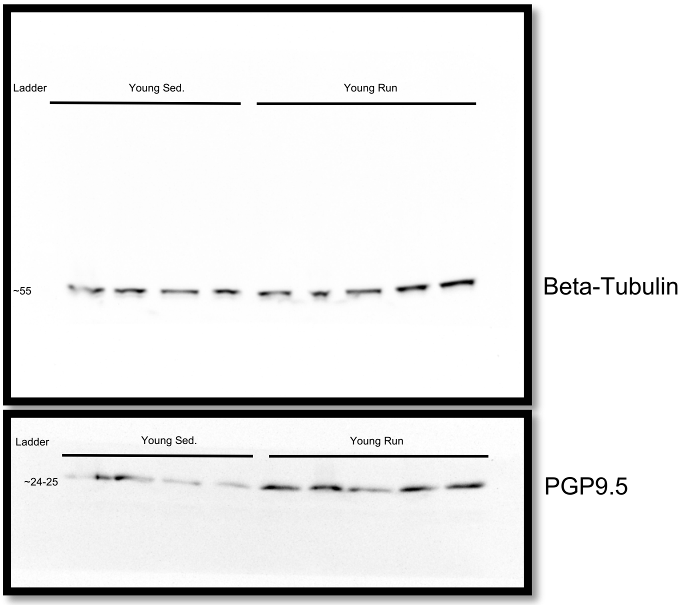

Blot associated with Fig 5b.

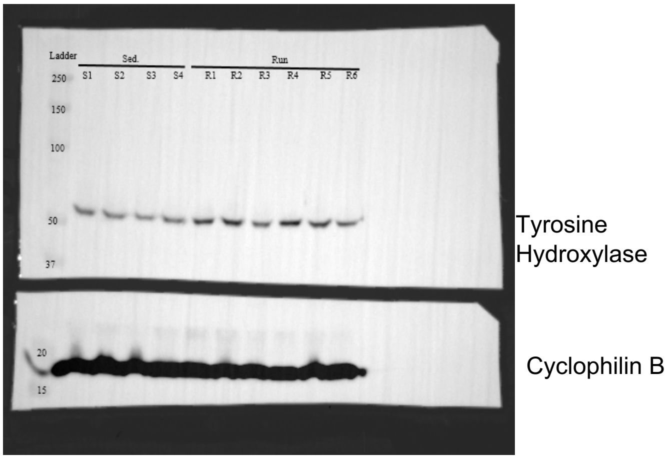

Blot associated with Fig 5c.

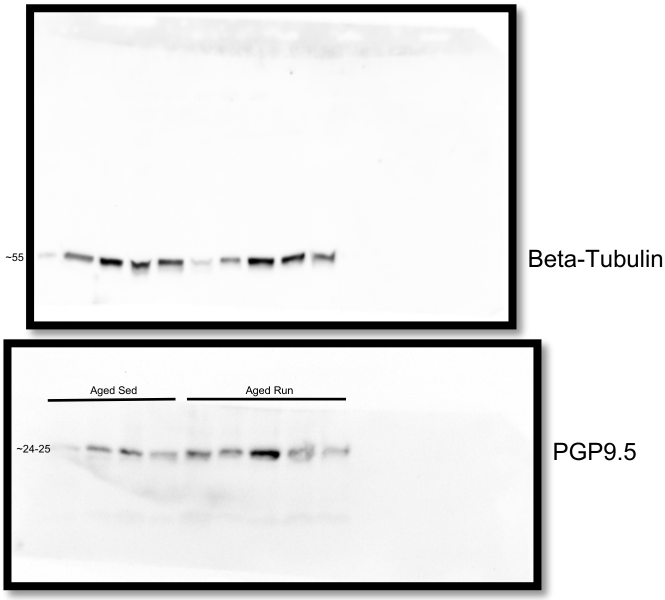

Blot associated with Fig 5d.

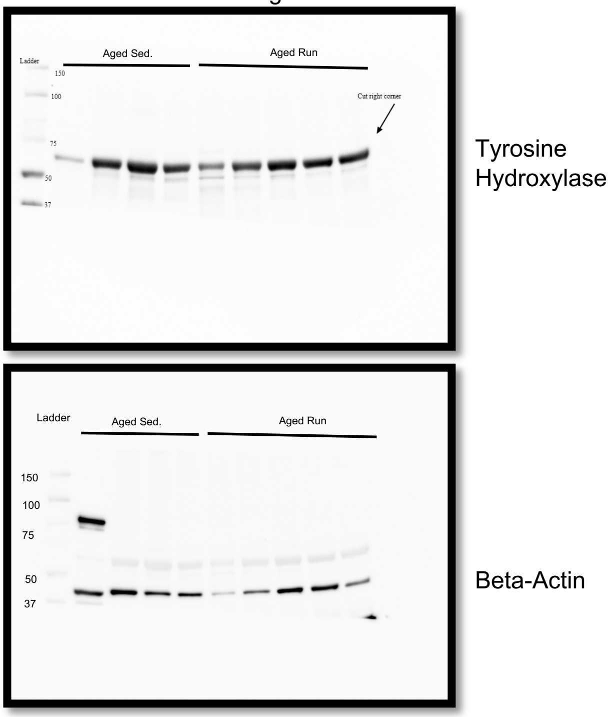

Blot associated with fig 5e.

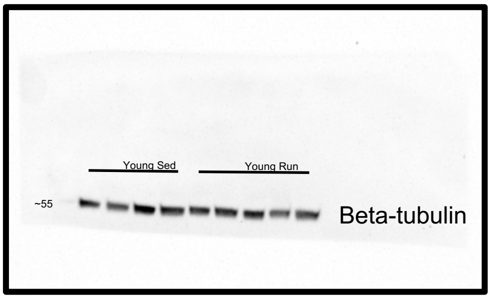

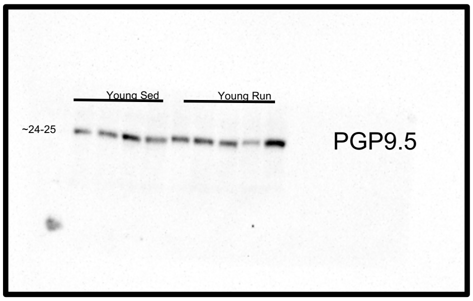

Blot associated with fig 5f.

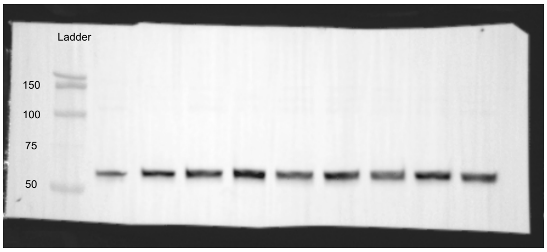

Tyrosine Hydroxylase

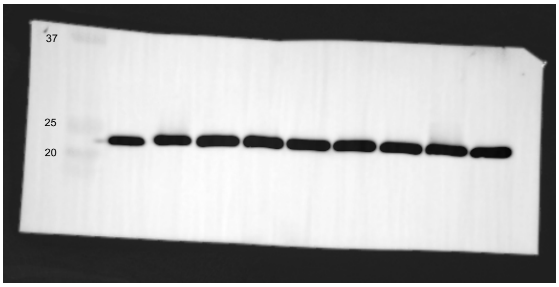

Cyclophilin B

Blot associated with fig 5g.

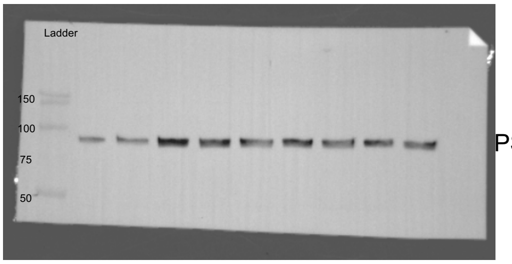

PSD95

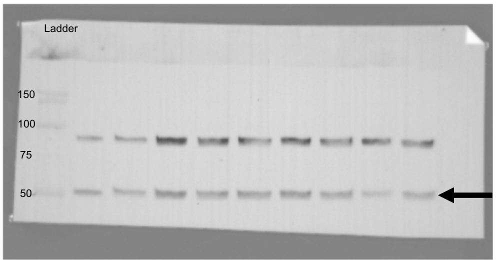

β-Tubulin

Blot associated with Fig 6a.

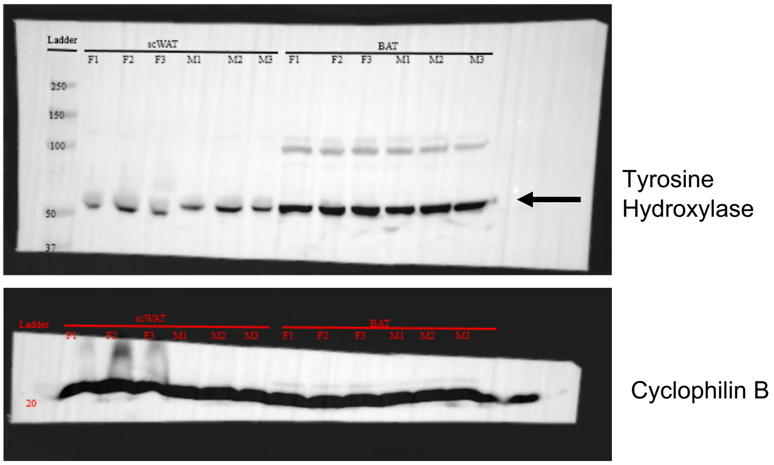

Blot associated with Fig 6b.

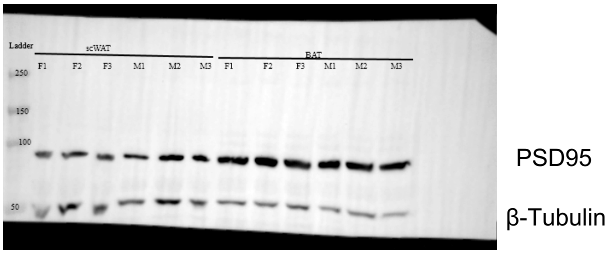

Blot associated with fig 6c.

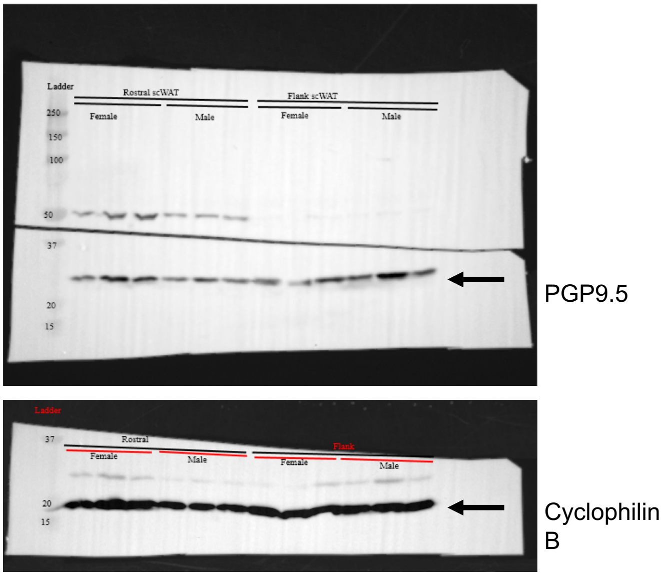

Blot associated with fig 6d.

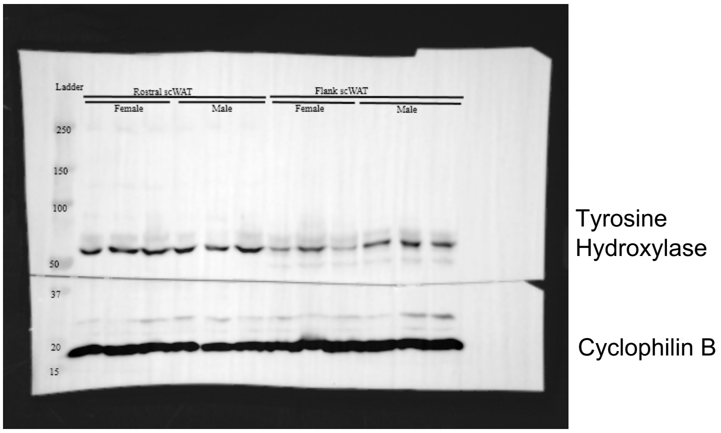

Blot associated with Fig 7a.

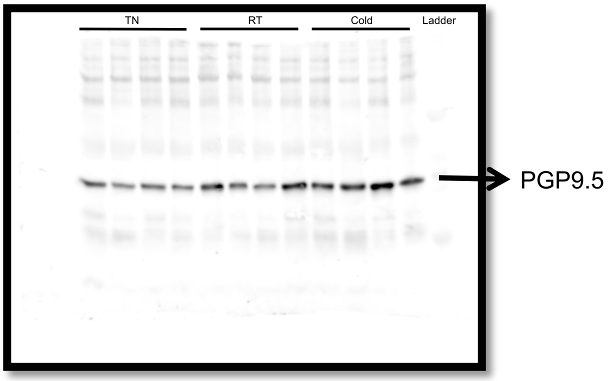

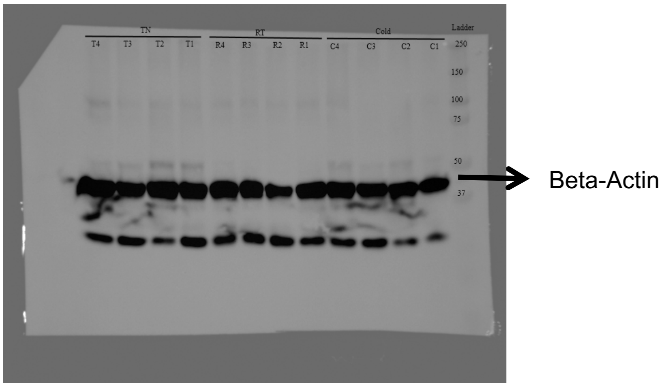

Blot associated with Suppl. Fig S1c.

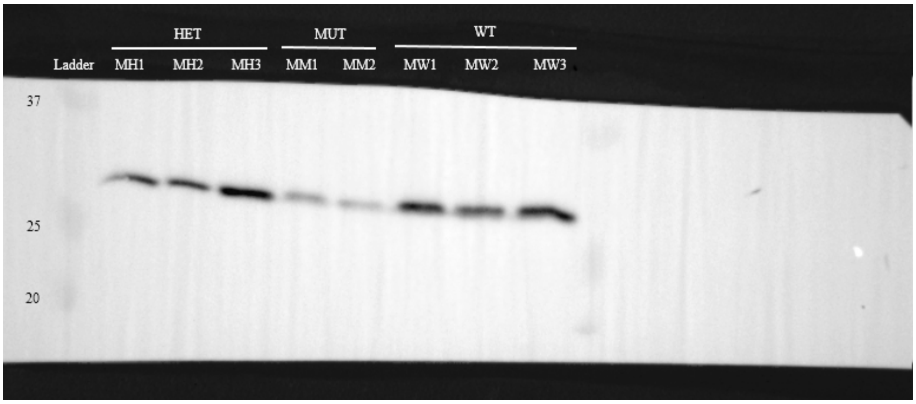

PGP9.5

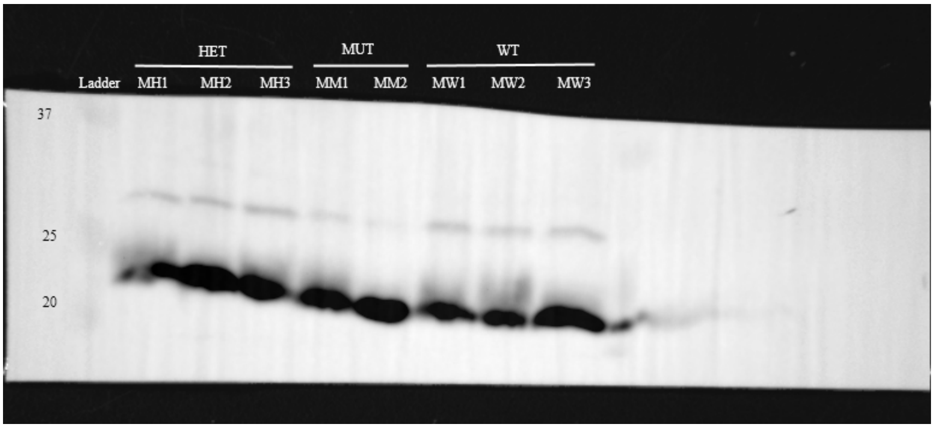

Cyclophilin B

Blot associated with Suppl. Fig S1d.

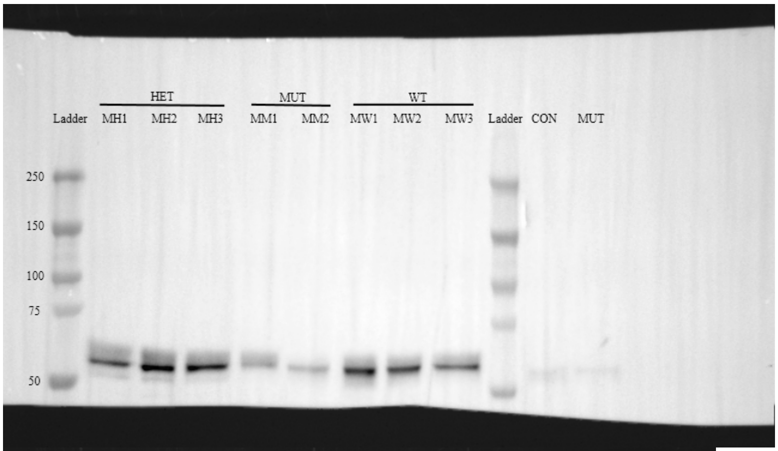

Tyrosine Hydroxylase

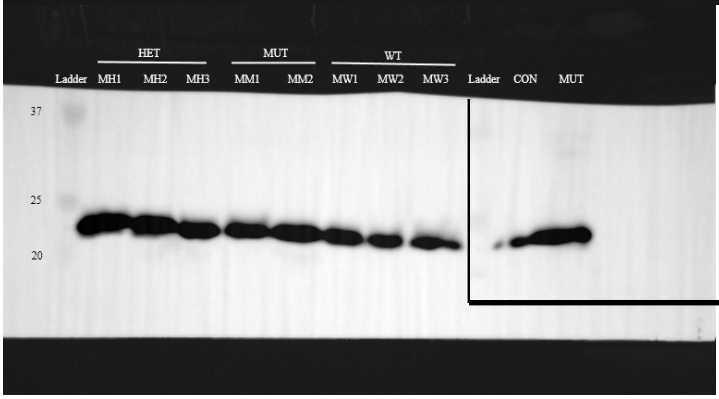

Cyclophilin B

Samples beyond second ladder not for this study

Blot associated with fig S2e.

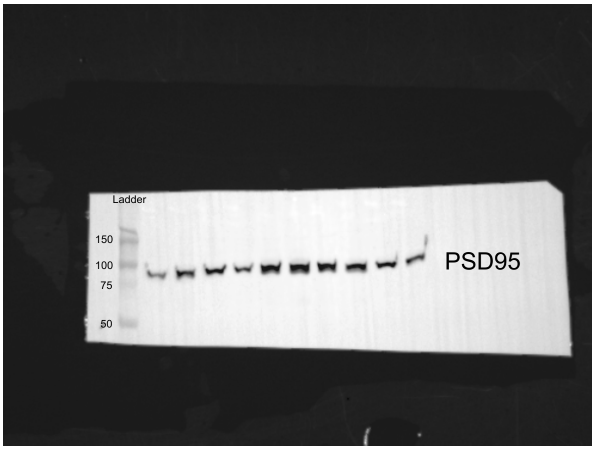

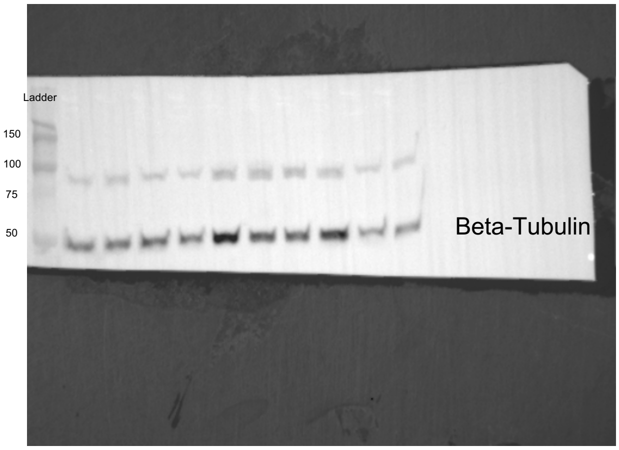

Supplement: S1 File — (PDF) [file pone.0221766.s007.pdf]
