# Supplementary figures and images for: Neuropathy and neural plasticity in the subcutaneous white adipose depot
Source: PLoS One. 2019 Sep 11;14(9):e0221766. doi: 10.1371/journal.pone.0221766 (PMC6738614; doi:10.1371/journal.pone.0221766)

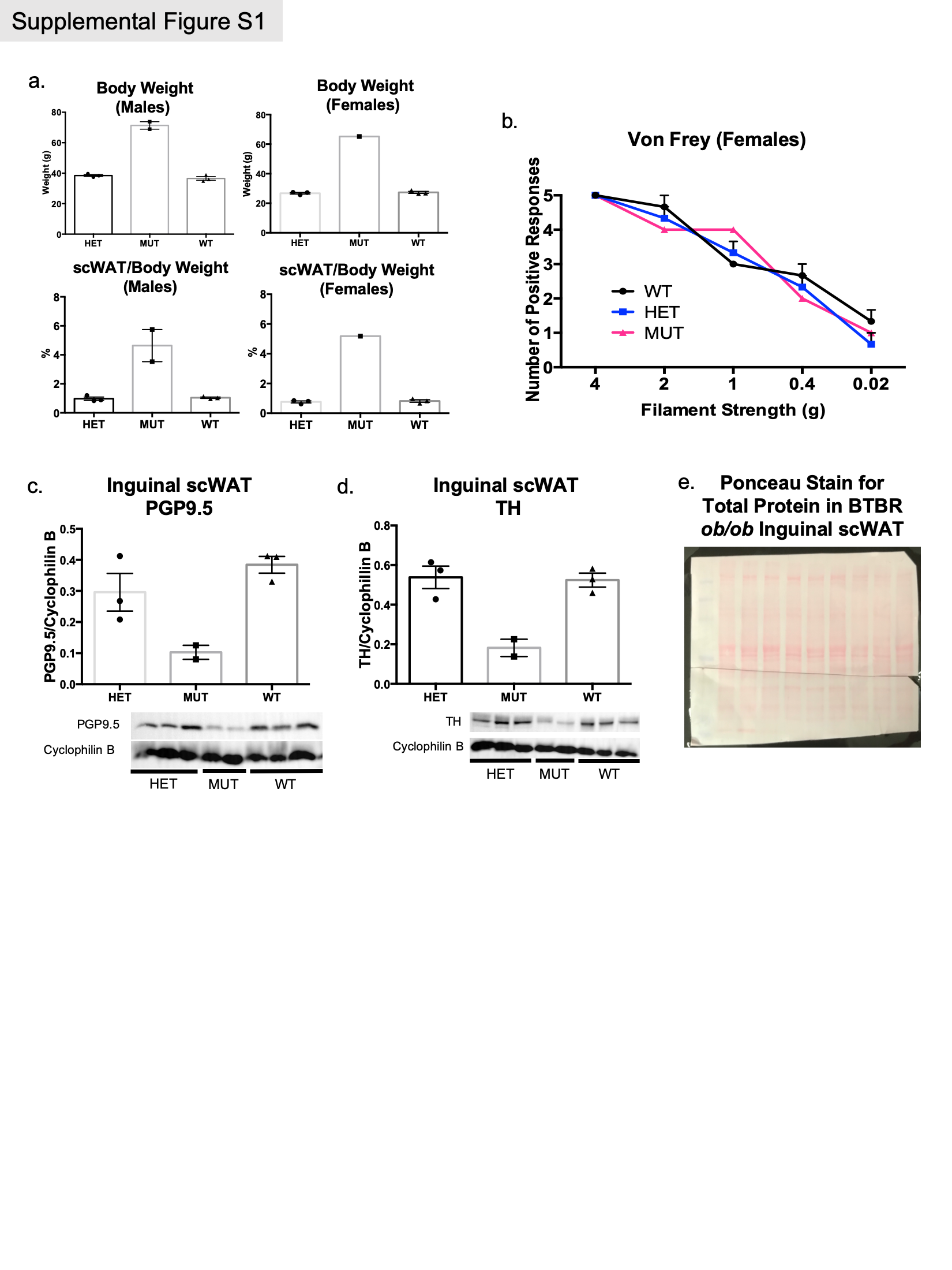

Supplement: S1 Fig — Male and female BTBR MUT mice were assessed for total body weight and adiposity (inguinal scWAT/body weight), and compared to WT or HET animals in a pilot cohort (a). Female BTBR WT, HET, and MUT were assessed for tactile allodynia via the Von Frey assay, an indirect measure of peripheral neuropathy (b). Protein expression of PGP9.5 (c) and TH (d) in inguinal scWAT of HET, MUT, and WT BTBR mice was measured via western blotting. Protein expression was normalized to Cyclophilin B and band density was quantified in Image J and analyzed using a two-tailed Student’s t-test. Error bars are SEMs. For (a-d), males: MUT N = 2; WT N = 3; HET N = 3; females: MUT: N = 1; WT: N = 3; HET: N = 3; all mice were 12–24 weeks old. Data represents a pilot cohort to compare HET to MUT mice, and males to females, thus statistical analyses were not performed due to small sample sizes. Sample Ponceau S staining on blot corresponding to Fig 1F demonstrates equal protein loading (e). (TIF) [file pone.0221766.s008.tif]

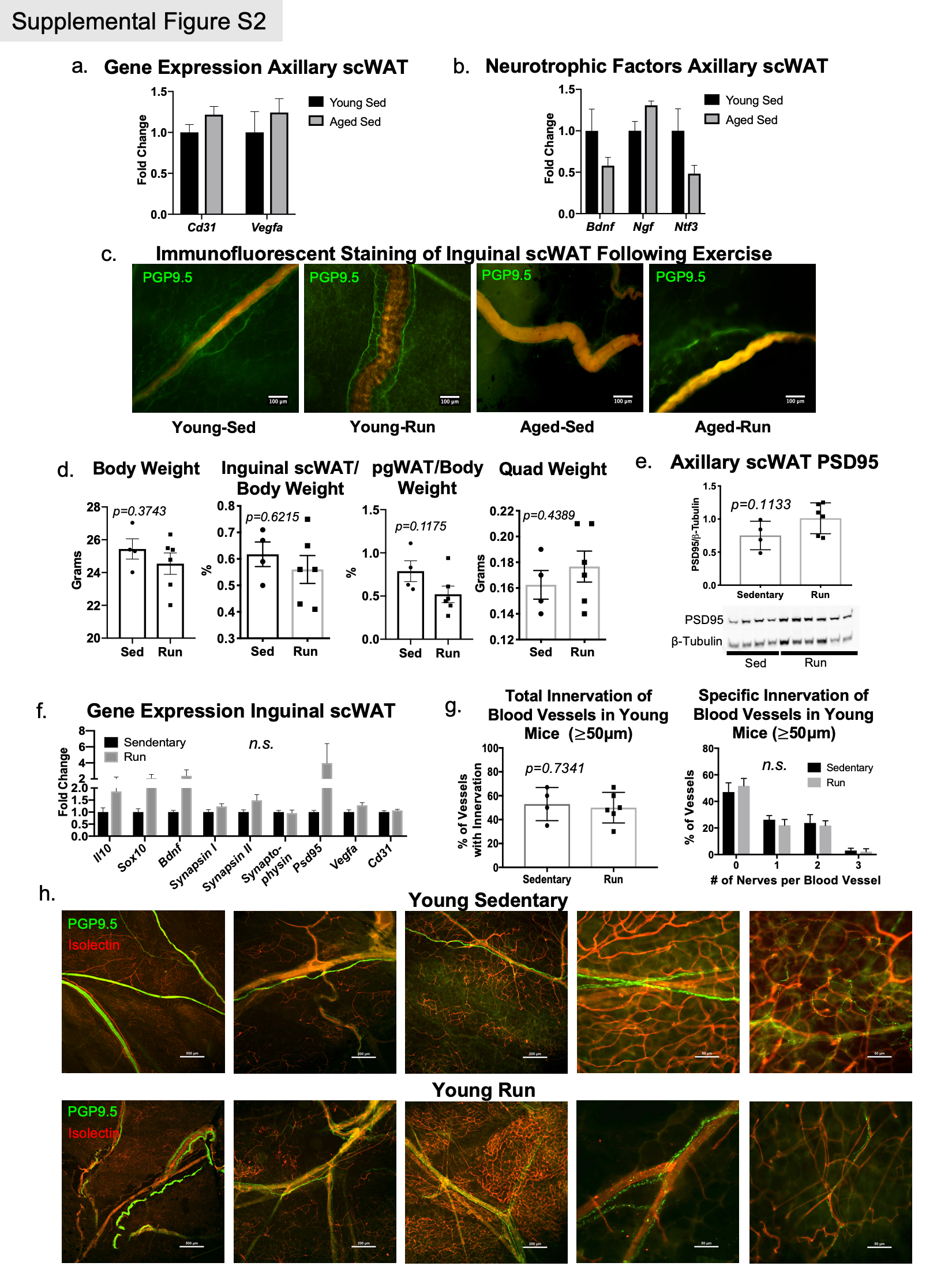

Supplement: S2 Fig — Gene expression analysis of axillary scWAT from young (10–12 week old) sedentary (young sed) versus aged (16 month old) sedentary (aged sed) male mice for vascular markers (a) and neurotrophic factors (b). Gene expression was analyzed by two-tailed Student’s t-test, N = 4 for sedentary and N = 5 for run. Error bars are SEMs. vascular markers. Whole depot nerve and vasculature imaging of inguinal scWAT from exercise (run) animals was performed by combining immunostaining for PGP9.5 (green) with autofluorescence of vasculature (red/orange); images were captured at 10x on Nikon Eclipse E400 microscope (c). Images are representative of N = 5 mice analyzed per group. Body weight, adiposity (inguinal scWAT/body weight & pgWAT/body weight), and quadricep muscle weight was measured for young (12–15 week old) mice under sedentary (sed) and exercised (run) conditions (d). Body and tissue weight analyzed by one-way ANOVA with Tukey post hoc. N = 4 for sedentary and N = 5 for run groups. Protein expression for PSD95 in axillary scWAT was determined by western blotting (e). Protein expression was normalized to β-Tubulin; band density was quantified in Image J and analyzed by two-tailed Student’s t-test. Gene expression analysis of inguinal scWAT from young (12–15 week old) sedentary (young sed) versus young exercised (young run) male mice (f), gene expression was analyzed by two-tailed Student’s t-test, N = 4 for sedentary and N = 5 for run. Error bars are SEMs. Whole inguinal scWAT depots were collected from young C57BL6/J mice under sedentary and exercised (run) conditions. Tissue was stained with PGP9.5 (green) and Isolectin IB-4 (red) to analyze nerve and blood vessel interactions in WAT with exercise. Tissues were scanned at 10x for blood vessels 50um or greater in diameter. Up to twenty blood vessels were evaluated for innervation in each tissue (g). Percentage of innervated vessels with a diameter of ≥ 50um were evaluated per tissue (g, left panel). Analyzed with two-tail [file pone.0221766.s009.tif]
